# Olive oil consumption, plasma metabolites, and risk of type 2 diabetes and cardiovascular disease

**DOI:** 10.1186/s12933-023-02066-1

**Published:** 2023-12-13

**Authors:** Jesús F. García-Gavilán, Nancy Babio, Estefanía Toledo, Zhila Semnani-Azad, Cristina Razquin, Courtney Dennis, Amy Deik, Dolores Corella, Ramón Estruch, Emilio Ros, Montserrat Fitó, Fernando Arós, Miquel Fiol, José Lapetra, Rosa Lamuela-Raventos, Clary Clish, Miguel Ruiz-Canela, Miguel Ángel Martínez-González, Frank Hu, Jordi Salas-Salvadó, Marta Guasch-Ferré

**Affiliations:** 1 Universitat Rovira i Virgili, Departament de Bioquímica i Biotecnologia, Alimentaciò, Nutrició Desenvolupament i Salut Mental ANUT-DSM, Reus, Spain; 2 Institut d’Investigació Sanitària Pere Virgili (IISPV), Reus, Spain; 3 CIBER de Fisiopatología de la Obesidad y Nutrición, Instituto de Salud Carlos III, Madrid, Spain; 4 Department of Preventive Medicine and Public Health, University of Navarra, IdiSNA, Pamplona, Spain; 5 Department of Nutrition, Harvard T.H. Chan School of Public Health, Boston, MA USA; 6 The Broad Institute of Harvard and MIT, Boston, MA USA; 7 Department of Preventive Medicine, University of Valencia, Valencia, Spain; 8 Department of Internal Medicine, Institut d’Investigacions Biomèdiques August Pi Sunyer (IDIBAPS), Hospital Clinic, University of Barcelona, Barcelona, Spain; 9 Institut de Nutrició I Seguretat Alimentària (INSA-UB), Universitat de Barcelona, Barcelona, Spain; 10 Department of Endocrinology and Nutrition, Lipid Clinic, IDIBAPS, Hospital Clinic, University of Barcelona, Barcelona, Spain; 11 Cardiovascular and Nutrition Research Group, Institut de Recerca Hospital del Mar, Barcelona, Spain; 12 Bioaraba Health Research Institute, Osakidetza Basque Health Service, Araba University Hospital, University of the Basque Country UPV/EHU, 01009 Vitoria-Gasteiz, Spain; 13 Plataforma de Ensayos Clínicos, Instituto de Investigación Sanitaria Illes Balears (IdISBa), Hospital Universitario Son Espases, 07120 Palma, Spain; 14 Department of Family Medicine, Research Unit, Distrito Sanitario Atención Primaria Sevilla, Seville, Spain; 15 Polyphenol Research Group, Departament de Nutrició, Ciències de L’Alimentació I Gastronomia, Universitat de Barcelon (UB), Av. de Joan XXII, 27-31, 08028 Barcelona, Spain; 16 Department of Epidemiology, Harvard T.H. Chan School of Public Health, Boston, MA USA; 17 Channing Division of Network Medicine, Department of Medicine, Brigham and Women’s Hospital and Harvard Medical School, Boston, MA USA; 18 Department of Public Health, Section of Epidemiology, Faculty of Health and Medical Sciences, University of Copenhagen, Copenhagen, Denmark; 19 Faculty of Health and Medical Sciences, Novo Nordisk Foundation Center for Basic Metabolic Research, University of Copenhagen, Øster Farimagsgade 5, 1014 Copenhagen, Denmark

**Keywords:** Olive oil, Metabolomics, Cardiovascular disease, Type 2 diabetes

## Abstract

**Background:**

Olive oil consumption has been inversely associated with the risk of type 2 diabetes (T2D) and cardiovascular disease (CVD). However, the impact of olive oil consumption on plasma metabolites remains poorly understood. This study aims to identify plasma metabolites related to total and specific types of olive oil consumption, and to assess the prospective associations of the identified multi-metabolite profiles with the risk of T2D and CVD.

**Methods:**

The discovery population included 1837 participants at high cardiovascular risk from the PREvención con DIeta MEDiterránea (PREDIMED) trial with available metabolomics data at baseline. Olive oil consumption was determined through food-frequency questionnaires (FFQ) and adjusted for total energy. A total of 1522 participants also had available metabolomics data at year 1 and were used as the internal validation sample. Plasma metabolomics analyses were performed using LC–MS. Cross-sectional associations between 385 known candidate metabolites and olive oil consumption were assessed using elastic net regression analysis. A 10-cross-validation (CV) procedure was used, and Pearson correlation coefficients were assessed between metabolite-weighted models and FFQ-derived olive oil consumption in each pair of training–validation data sets within the discovery sample. We further estimated the prospective associations of the identified plasma multi-metabolite profile with incident T2D and CVD using multivariable Cox regression models.

**Results:**

We identified a metabolomic signature for the consumption of total olive oil (with 74 metabolites), VOO (with 78 metabolites), and COO (with 17 metabolites), including several lipids, acylcarnitines, and amino acids. 10-CV Pearson correlation coefficients between total olive oil consumption derived from FFQs and the multi-metabolite profile were 0.40 (95% CI 0.37, 0.44) and 0.27 (95% CI 0.22, 0.31) for the discovery and validation sample, respectively. We identified several overlapping and distinct metabolites according to the type of olive oil consumed. The baseline metabolite profiles of total and extra virgin olive oil were inversely associated with CVD incidence (HR per 1SD: 0.79; 95% CI 0.67, 0.92 for total olive oil and 0.70; 0.59, 0.83 for extra virgin olive oil) after adjustment for confounders. However, no significant associations were observed between these metabolite profiles and T2D incidence.

**Conclusions:**

This study reveals a panel of plasma metabolites linked to the consumption of total and specific types of olive oil. The metabolite profiles of total olive oil consumption and extra virgin olive oil were associated with a decreased risk of incident CVD in a high cardiovascular-risk Mediterranean population, though no associations were observed with T2D incidence.

*Trial registration***:** The PREDIMED trial was registered at ISRCTN (http://www.isrctn.com/, ISRCTN35739639).

**Supplementary Information:**

The online version contains supplementary material available at 10.1186/s12933-023-02066-1.

## Introduction

Olive oil has been traditionally used as the main culinary and dressing fat in Mediterranean regions and is gaining global popularity due to its remarkable nutritional profile and health benefits. It has been proposed as one of the key components of the Mediterranean Diet (MedDiet), which makes it cardio-protective [1]. The best nutritional and organoleptic quality olive oil, extra-virgin olive oil, and virgin olive oil (VOO) varieties are obtained by mechanically pressing olives. They contain an exceptional matrix of lipids rich in monounsaturated fatty acids (mainly oleic acid) and high amounts of bioactive compounds, including polyphenols (hydroxytyrosol and oleuropein), lipid derivatives (squalene, tocopherols), and vitamin E, and have a richer taste, color, and aroma than other common varieties. Because of its processing, the refined or common variety of olive oil (COO) has a similar lipid profile but a lower content of phytochemicals [2]. Evidence from epidemiological studies and clinical trials support that olive oil consumption is associated with a lower risk of type 2 diabetes (T2D), cardiovascular diseases (CVD), and mortality [3–5], and has demonstrated protective effects on lipid metabolism, inflammation, endothelial function, and oxidative stress [6–8]. Despite the mounting evidence supporting olive oil’s beneficial impact on human health, the lack of detailed differentiation between olive oil types in existing studies is a significant limitation. This oversight hinders a comprehensive understanding of the distinctive health properties offered by VOO.

Despite the wide range of health benefits attributed to olive oil consumption, the biological mechanisms underlying these salutary effects have not been well defined. Nutritional metabolomics, an evolving approach, holds great promise in enhancing our understanding of the biological effects of nutritional factors and may also help to identify potential novel biomarkers related to dietary intake or the subsequent metabolic response to this intake. Along these lines, the distinct phytochemical concentrations found in olive oil varieties may confer specific health advantages [7]. For example, VOO consumption has been associated with better HDL functionality, indicating a potential mediating role between metabolic pathways associated with cardiovascular health and VOO consumption [9, 10]. We hypothesized that the amount of total olive oil consumed, and specifically if the consumption is mainly from VOO or common olive oil, is associated with unique metabolite profiles and that these multi-metabolite profiles are related to a lower incidence of T2D and CVD.

In the current study, leveraging dietary and metabolomics data in the PREvención con DIeta MEDiterránea (PREDIMED) study, we used an agnostic machine learning approach to identify plasma metabolite profiles associated with total olive oil, VOO, and COO. We then evaluated whether the identified multi-metabolite profiles are associated with T2D and CVD incidence risk independently of known risk factors and diet.

## Methods

### Study population

The present analysis was conducted in the context of the PREDIMED study. This study was a Spanish multicenter randomized controlled nutritional intervention trial conducted between 2003 and 2010. The main objective was to examine the effect of the MedDiet on the primary prevention of CVD in a population with several risk factors for CVD. The full protocol of the PREDIMED study has been previously published and can be found on the study website (http://www.predimed.es/) [11, 12]. Study participants provided written informed consent and all the study centers have approved the protocol by their Institutional Review Boards. The PREDIMED trial is registered at ISRCTN (http://www.isrctn.com/ ISRCTN35739639).

The participants involved in this analysis are from three nested study samples: the first study for T2D (the PREDIMED-T2D study), the second study for CVD (the PREDIMED-CVD study), and a third subset of PREDIMED participants who completed an oral glucose tolerance test (OGTT) at baseline. The first case-cohort study consisted of 251 participants with incident T2D cases and 694 participants without T2D at baseline (overlapping participants n = 53 between cases and cohort participants) [13, 14]. The second case-cohort study consisted of 229 participants with incident CVD cases (a composite of myocardial infarction, stroke, and CVD mortality) and 788 participants without CVD at baseline (overlapping n = 37 between cases and cohort participants). The third study consisted of 132 participants. More information about these studies is available elsewhere [15, 16].

Participants who had complete metabolomics data and nutritional data at baseline (from semi-quantitative food frequency questionnaires (FFQs)) and were not duplicated (overlapping participants between databases from different metabolomics sub-studies, n = 122) were selected (n = 1,882). Additionally, participants with missing values in FFQs at baseline (n = 11), a daily energy intake lower than 500 kcal for women or 800 kcal for men and higher than 3500 kcal for women and 4000 kcal for men (n = 30) [17], or with missing values in ≥ 20% metabolites (n = 4) were excluded. Therefore, this analysis included 1,837 participants at baseline (634 participants allocated to the MedDiet supplemented with VOO group, 630 to the MedDiet supplemented with nuts, and 573 participants to the control group) (Additional file 1: Figure S1).

Further, an internal validation in the same population was conducted using dietary and metabolomics data from the 1-year visit in 1522 study participants (Additional file 1: Figure S1).

### Dietary assessment

Dietary data were obtained using a validated 137-item semi-quantitative FFQ that trained dietitians collected from the participants at baseline and 1-year visits in face-to-face interviews [18]. Food, nutrients, and energy intake were estimated using Spanish food composition tables [19, 20]. Total olive oil, VOO, and COO consumption were derived from these FFQs. Total olive oil consumption was considered the sum of VOO and COO.

### Anthropometric measurements and other covariates

At baseline and 1-year visits, blood pressure (in triplicate) and anthropometric measurements such as weight, height, and waist circumference were measured according to the study protocol by trained staff. Additionally, physical activity was assessed with the validated Spanish version of the Minnesota Leisure-Time Physical Activity questionnaire [21] and other information about lifestyle, medical conditions, or medication use was also collected.

### Metabolite profiling

At baseline and 1-year visits, overnight fasting plasma EDTA samples (> 8 h) were collected, processed, and stored in -80◦C freezers at each recruiting center. Before metabolomics assays, case-cohort participant samples were randomized in pairs (baseline plus 1-year visit) and sent to the Broad Institute of Harvard University and the Massachusetts Institute of Technology for analysis. Metabolic profiling of the plasma samples was performed using high-throughput liquid chromatography-tandem mass spectrometry (LC–MS) techniques [22]. After quality filtration and standardization, 400 known metabolites were quantified, of which 19 metabolites were removed from the analyses (3 metabolites that were considered as internal standards (1,2-didodecanoyl-sn-glycerol-3-phosphocholine, valine-d8, and phenylalanine-d8), 7 metabolites that were drugs (acetaminophen, metronidazole, metformin, valsartan, warfarin, verapamil, atenolol) and 9 metabolites due to > 20% of missing values). The analyses were conducted with 381 known metabolites.

To quantitatively profile polar metabolites and plasma lipids, LC–MS was used as previously described [23–25]. Amino acids (AAs) and other polar metabolites were profiled with a Nexera X2 U-HPLC (Shimadzu Corp., Marlborough, MA) coupled to a Q-Exactive mass spectrometer (ThermoFisher Scientific, Waltham, MA). Metabolites were extracted from 10 μL plasma and 90 μL of acetonitrile/methanol/formic acid (74.9:24.9:0.2 vol:vol:vol) that contained stable isotope-labeled internal standards [valine-d8 (Sigma-Aldrich) and phenylalanine-d8 (Cambridge Isotope Laboratories)]. After centrifuging at 9,000 × g for 10 min at 4◦C, the samples supernatants were injected directly onto a 150 × 2-mm, 3-μm Atlantis HILIC column (Waters). The column was eluted isocratically at a flow rate of 250 L/min with 5% mobile phase A (10 mmol ammonium formate/L and 0.1% formic acid in water) for 0.5 min followed by a linear gradient to 40% mobile phase B (acetonitrile with 0.1% formic acid) over 10 min. MS analyses were carried out using electrospray ionization in the positive-ion mode. Full-scan spectra were acquired over 70–800 m/z. Fatty acids and other lipids were also profiled using a Nexera X2 U-HPLC (Shimadzu Corp., Marlborough, MA) coupled to an Exactive Plus orbitrap MS (Thermo Fisher Scientific) and were extracted from 10 μL plasma using 190 μL of isopropanol containing 1,2-didodecanoyl-sn-glycerol-3-phosphocholine (Avanti Polar Lipids) as an internal standard. The lipid extraction (2 μL) was injected into a 100 × 2.1-mm, 1.7-μm ACQUITY BEH C8 column (Waters). The column was eluted isocratically with 80% mobile-phase A of (95:5:0.1 vol:vol:vol 10 mM ammonium acetate/methanol/formic acid) for 1 min followed by a linear gradient to 80% mobile-phase B (99.9:0.1 vol:vol methanol/formic acid) over 2 min, a linear gradient to 100% mobile-phase B over 7 min, and then 3 min at 100% mobile-phase B. For the AAs, MS analyses were carried out using electrospray ionization in the positive-ion mode using full-scan analysis over 200–1100 m/z. Raw data were processed using Trace Finder version 3.1 and 3.3 (Thermo Fisher Scientific) and Progenesis QI (Nonlinear Dynamics). Polar metabolite identities were confirmed using authentic reference standards. Lipids were identified using the head group, total acyl carbon numbers, and total acyl double bond content. Pairs of pooled plasma reference samples were analyzed in intervals of 20 participant samples to assess data quality and to facilitate data standardization across the analytical queue and sample batches. One sample of each pair of the pooled references functioned as a passive quality control to assess the analytical measurement reproducibility of each metabolite. The other pooled sample was used to standardize using a “nearest neighbor” approach, i.e., standardized values were calculated using the ratio of the value in each sample over the nearest pooled plasma reference multiplied by the median value measured across the pooled references.

### Statistical analysis

Baseline characteristics were presented as means and standard deviations (SD) when variables were quantitative and as percentages (n) when variables were categorical. The metabolites associated with total olive oil, VOO, and COO consumption were selected using plasma baseline metabolomics data (i.e., discovery sample). Plasma metabolomics data at 1 year were used as validation samples. Total olive oil, VOO, and COO consumption at baseline and 1 year were adjusted for total energy intake using the residual method [17].

The statistical quality controls used with the metabolomics data were as follows: When metabolites presented missing values of less than 20% (i.e., not detectable/quantifiable concentrations or not present metabolite), they were imputed using a random forest approach ("missForest" function from the "missForest" R package) as previous publications have recommended [26–28]. Metabolites were normalized and scaled using Blom's rank-based inverse normal transformation [29].

Linear regression models were used to assess associations between plasma baseline metabolites and total olive oil, VOO, and COO consumption. Models were adjusted for recruiting center, age, sex, smoking status (former smoker, never smoker, smoker), BMI, education, and physical activity (METs-min/day). Multiple testing correction was performed using the Benjamini–Hochberg procedure (FDR) and reported findings with FDR *p*-Adjusted value < 0.05.

To determine the metabolomic profile associated with each exposure (total olive oil, VOO, and COO) using an agnostic metabolomics approach including knowing metabolites, Gaussian linear regression models were used with the elastic net penalty (ENR) (“caret” v 6.0–84 and “glmnet” R Package). A tenfold cross-validation (CV) approach was performed with the discovery population (PREDIMED baseline data). First, the sample was split into 10 training-validation sets (90–10%, respectively) and we performed a tenfold CV to find the optimal value of the tuning parameter (λ) that results in a mean squared error within 1 SD of the minimum (minMSE). Additionally, the α parameter was evaluated from 0 (i.e., a Ridge regression) to 1 (i.e., a Lasso regression) in 0.1 increments to test the best parameters for these analyses. The best-predicting accuracy in the validation sets was obtained with α = 0.4 for total olive oil, 0.2 for VOO, and 0.3 for COO models. After evaluating the tuning parameters, a tenfold CV was performed again with the discovery population, the coefficients from each tenfold CV iteration were extracted and were constructed weighted models using the regression coefficients of the selected metabolites from each ENR in the training set to validate with its validation data set pair. Pearson correlation coefficients were determined between total olive oil, VOO, and COO consumption and the metabolomics profile in the pair of training validation data sets in both the discovery and internal validation samples (i.e., PREDIMED baseline data and PREDIMED 1-year data). For reproducibility, regression coefficients were reported using 10 iterations of the tenfold CV elastic net regression in the entire data set. These analyses were based on consistency among CV runs; therefore, any *P*-value was derived.

The associations between the identified metabolite profiles of total olive oil, VOO, and COO consumption (1 SD) and T2D risk (245 events at baseline and 161 incident events at 1 year) within the T2D nested case-cohort study and CVD risk (222 events at baseline and 159 incident events at 1 year) within the CVD nested case-cohort study were run with Cox regressions with Barlow weights and robust variance estimator. Four multivariate models were assessed. For the baseline analysis, the first model was adjusted for age (years), sex, and propensity scores, as previously described [12], and was stratified by intervention group and recruitment center. The second model was additionally adjusted for BMI (kg/m^2^), smoking status (current, former, or never), alcohol consumption in g/day (and adding a quadratic term), educational level (primary, secondary, or college), physical activity (METs/min/day), family history of CVD (yes/no), the baseline prevalence of dyslipidemia and hypertension, and dyslipidemia and hypertension medication. The third model included all covariables from model 2 in addition to the consumption of vegetables, fruits, cereals, nuts, eggs, legumes, fish, meat, and dairy products (all g/day). The fourth model included all covariates from model 3 in addition to the baseline consumption of total olive oil, VOO, or COO from which the metabolite set was derived, respectively. For the 1-year analyses, we used the same models as at baseline excluding those T2D or CVD cases diagnosed during the first year. Interactions between olive oil profiles and intervention groups were evaluated using the likelihood ratio test including the interaction product terms as covariables.

Several sensitivity analyses were conducted. First, we performed a principal component analysis (PCA) using the mean ENR coefficients from the metabolites consistently selected (i.e., 10 times) in each olive oil profile. A zero value was assigned whenever a particular metabolite was not found by a specific approach. Coefficients were centered and scaled before PCA analysis. Second, we evaluated the specificity of each metabolite profile using Pearson correlations between the consumption of each type of olive oil with each metabolomics profile. Third, we conducted stratified analyses by intervention group. Fourth, we further adjusted the multi-variable Cox regression models for coffee and tea intake.

All analyses were considered statistically significant when *P* < 0.05 and were performed using R version 4.2.2 statistical software (R Foundation for Statistical Computing).

## Results

### Characteristics of the study participants

The baseline characteristics of the study participants according to tertiles of energy-adjusted total olive oil consumption are shown in Table 1. The mean ± SD consumption of total olive oil at baseline was 22 ± 8 g/day in the lowest tertile compared to 56 ± 10 g/day in the highest tertile. For VOO and COO, respectively, the mean consumption was 9 ± 11 g/day and 12 ± 12 g/day in the first tertile, and 36 ± 27 g/day and 19 ± 25 g/day in the third tertile. In the top tertile, a higher percentage of participants had T2D, were less likely to smoke, and consumed lower amounts of fruits, vegetables, legumes, cereals, meat, and wine, as compared to participants in the lowest tertile.

**Table 1:** Baseline characteristics of study participants according to tertiles of energy-adjusted total olive oil consumption

	Tertile 1 (n = 613)	Tertile 2 (n = 612)	Tertile 3 (n = 612)
Age (years)	67 ± 6	67 ± 6	67 ± 6
Women, n (%)	339 (55)	363 (59)	356 (58)
Body mass index (kg/m^2^)	30 ± 3	30 ± 4	30 ± 4
Waist circumference (cm)	101 ± 10	100 ± 10	101 ± 10
Type 2 diabetes, n (%)	178 (29)	165 (27)	192 (31)
Hypercholesterolemia, n (%)	485 (79)	464 (76)	462 (76)
Hypertension, n (%)	531 (87)	540 (88)	532 (87)
Family history of CVD, n (%)	152 (25)	145 (24)	154 (25)
Current smoking, n (%)	103 (17)	101 (17)	83 (14)
Vegetables (g/day)	343 ± 164	323 ± 149	328 ± 136
Fruit (g/day)	374 ± 215	356 ± 183	352 ± 193
Legumes (g/day)	22 ± 16	20 ± 11	19 ± 10
Cereals (g/day)	249 ± 104	236 ± 106	208 ± 88
Dairy (g/day)	402 ± 235	376 ± 222	348 ± 202
Total meat (g/day)	139 ± 60	136 ± 55	127 ± 52
Total fish (g/day)	99 ± 50	104 ± 64	101 ± 43
Total olive oil (g/day)	22 ± 8	40 ± 12	56 ± 10
Virgin olive oil (g/day)	9 ± 11	21 ± 20	36 ± 27
Common olive oil (g/day)	12 ± 12	18 ± 19	19 ± 25
Nuts (g/day)	12 ± 14	11 ± 14	10 ± 13
Wine (g/day)	70 ± 115	75 ± 132	55 ± 99
Alcohol (g/day)	10 ± 16	11 ± 18	8 ± 12
Total energy (kcal/day)	2297 ± 489	2309 ± 670	2243 ± 450
Adherence to the MedDiet	8 ± 2	9 ± 2	9 ± 2

Values are means ± standard deviation for continuous variables or number (%) for categorical variables

*CVD* Cardiovascular disease, *MedDiet* Mediterranean diet

Total olive oil consumption was adjusted for total energy intake using the residual method

### Identification of metabolites associated with olive oil consumption

**Fig. 1:**
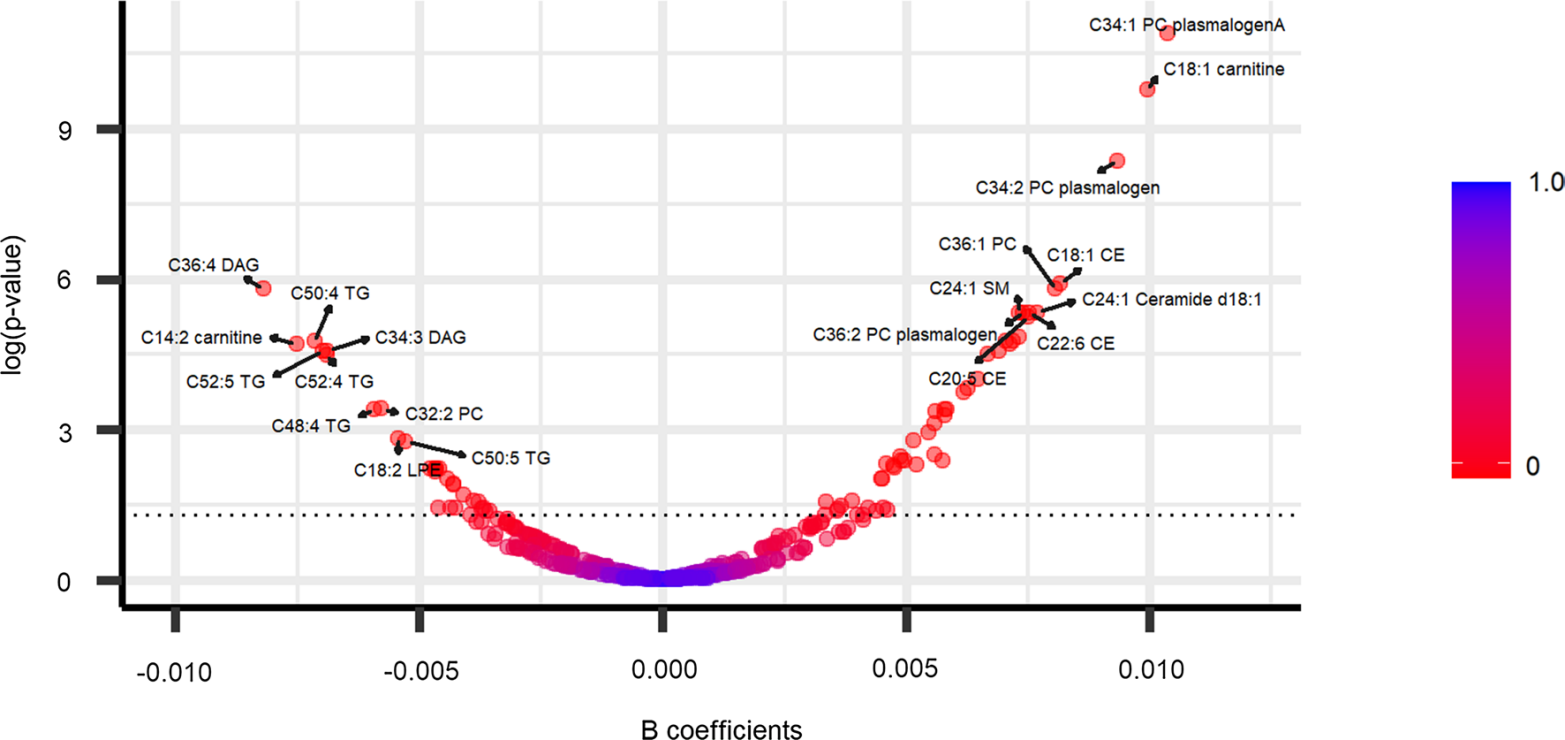
Volcano plot showing the associations between plasma metabolites and total olive oil consumption at baseline. The models were adjusted by recruiting center, smoking status (former smoker, never smoker, smoker), sex, BMI, age, education, and physical activity (METs/day). An FDR < 0.05 was considered statistically significant (up dotted line)

**Fig. 2:**
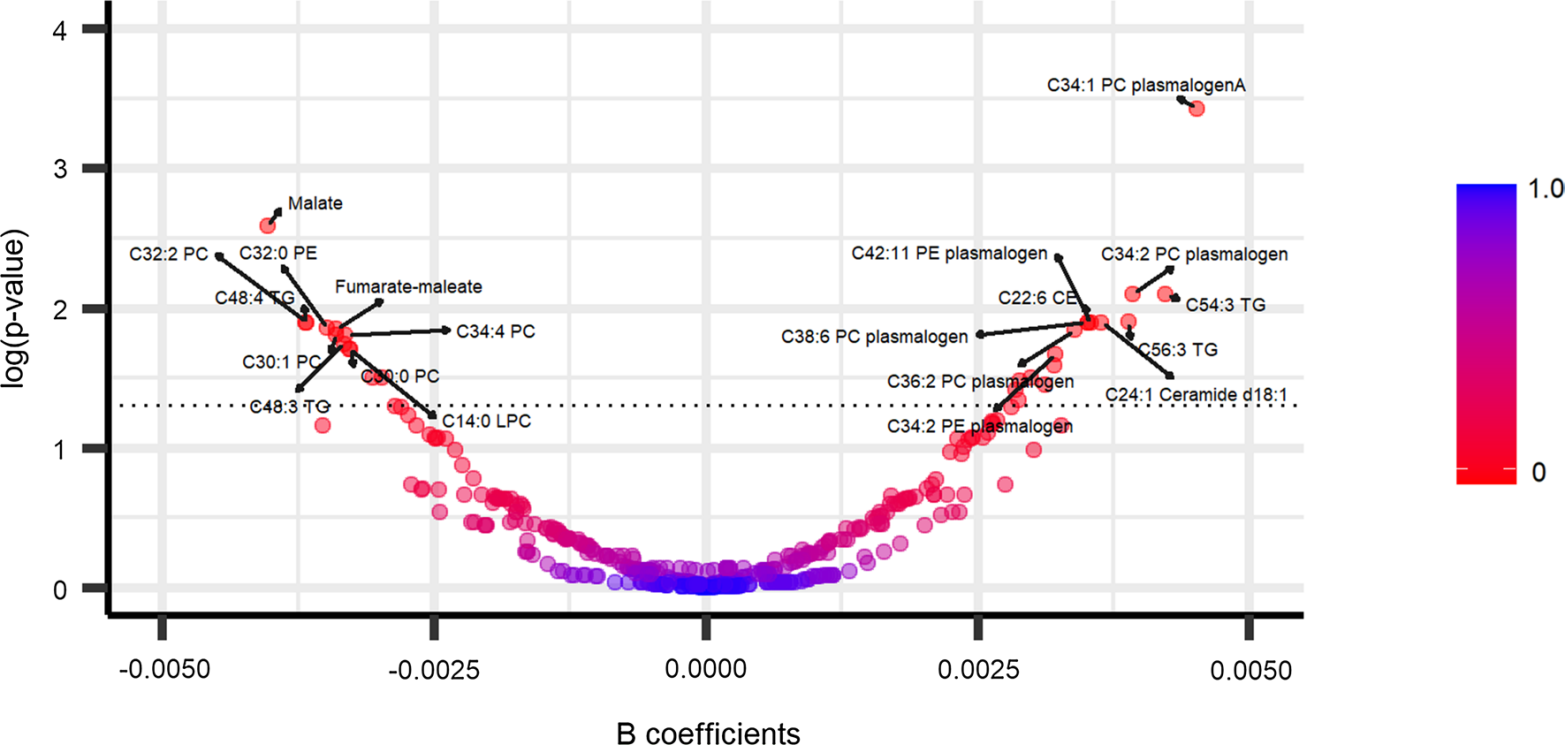
Volcano plot showing the associations between plasma metabolites and virgin olive oil consumption at baseline. The models were adjusted by recruiting center, smoking status (former smoker, never smoker, smoker), sex, BMI, age, education, and physical activity (METs/day). An FDR < 0.05 was considered statistically significant (up dotted line)

Cross-sectional associations between baseline plasma metabolites and baseline total olive oil and VOO consumption are shown in Figs. 1 and 2, and Additional file 1: Table S1. Associations between baseline plasma metabolites and baseline common olive oil consumption are shown in Additional file 1: Figure S2 and Additional file 1: Table S1. 78 metabolites were individually significantly associated with total olive oil consumption, 28 metabolites were significantly associated with VOO consumption, and 5 metabolites were significantly associated with COO consumption (FDR < 0.05).

**Fig. 3:**
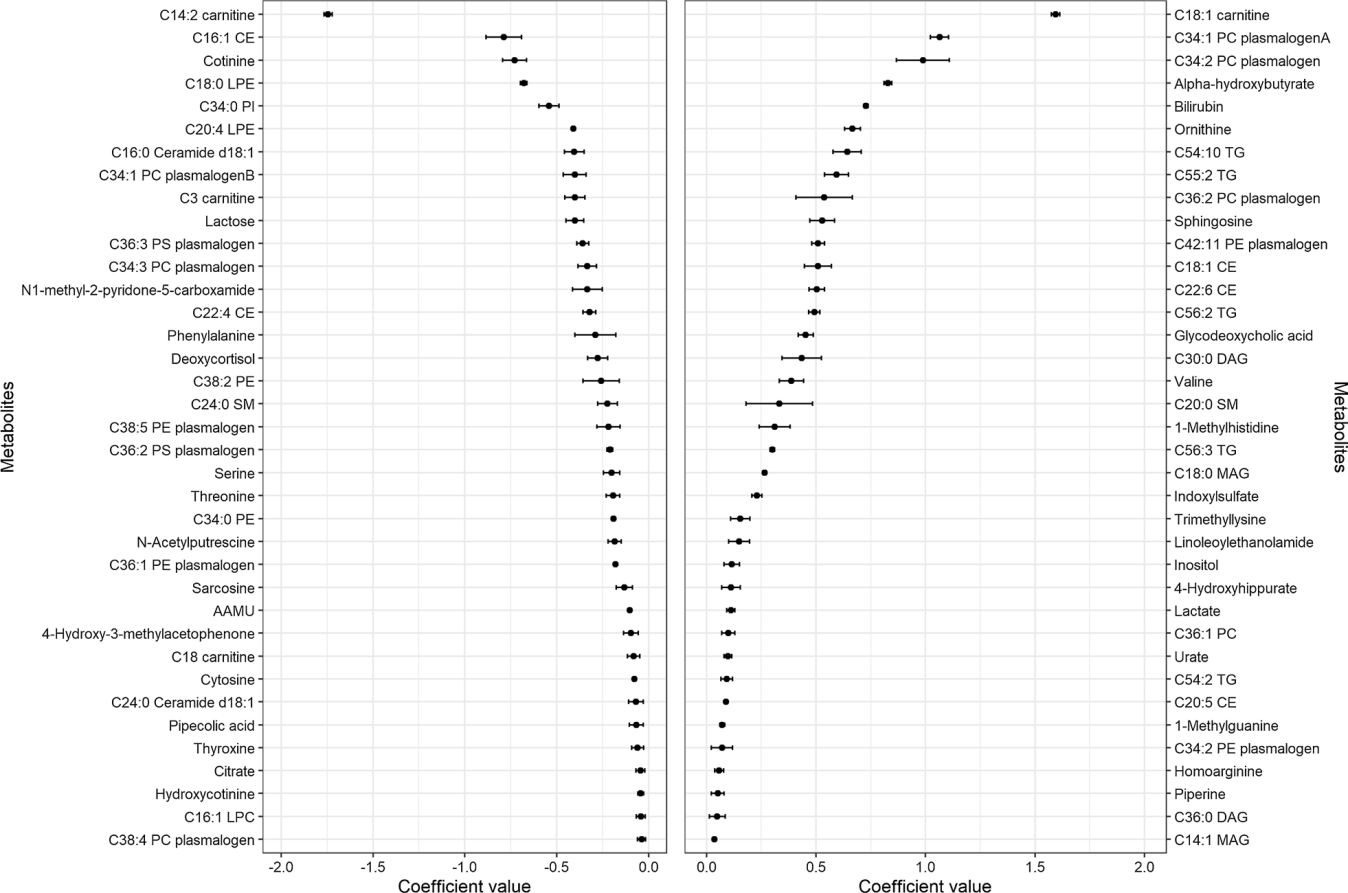
Coefficients (mean and SD) for the metabolites were selected ten times in the 10-cross validation of the continuous elastic regression for energy-adjusted total olive oil consumption. The sets of metabolites were selected using elastic continuous regression models (with lambda.min) employing the whole dataset of subjects (n = 1833). Negative coefficients are plotted on the left, whereas positive coefficients are shown on the right

**Fig. 4:**
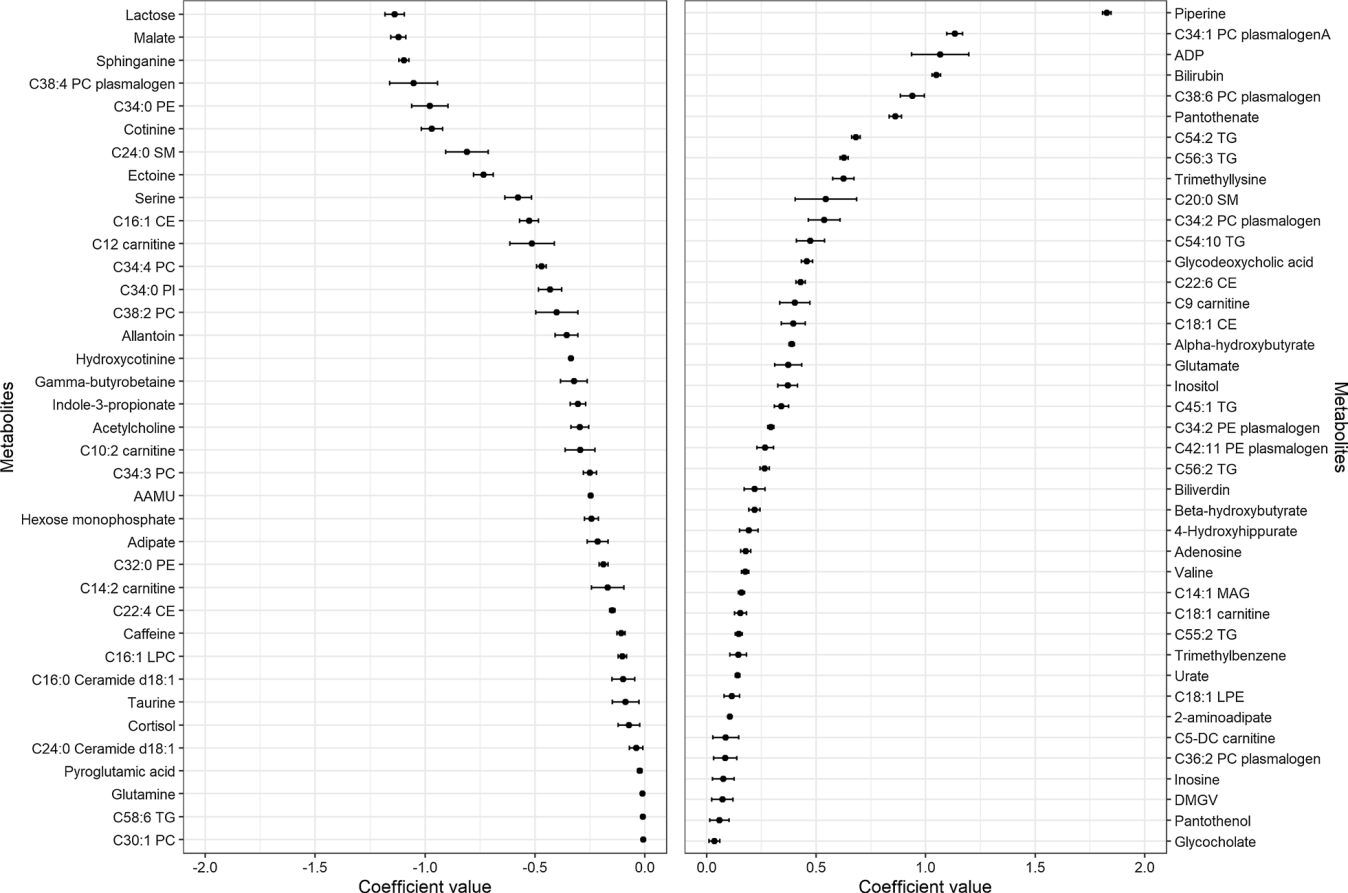
Coefficients (mean and SD) for the metabolites were selected ten times in the 10-cross validation of the continuous elastic regression for energy-adjusted virgin oil. The sets of metabolites were selected using elastic continuous regression models (with lambda.min) employing the whole dataset of subjects (n = 1833). Negative coefficients are plotted on the left, whereas positive coefficients are shown on the right

**Fig. 5:**
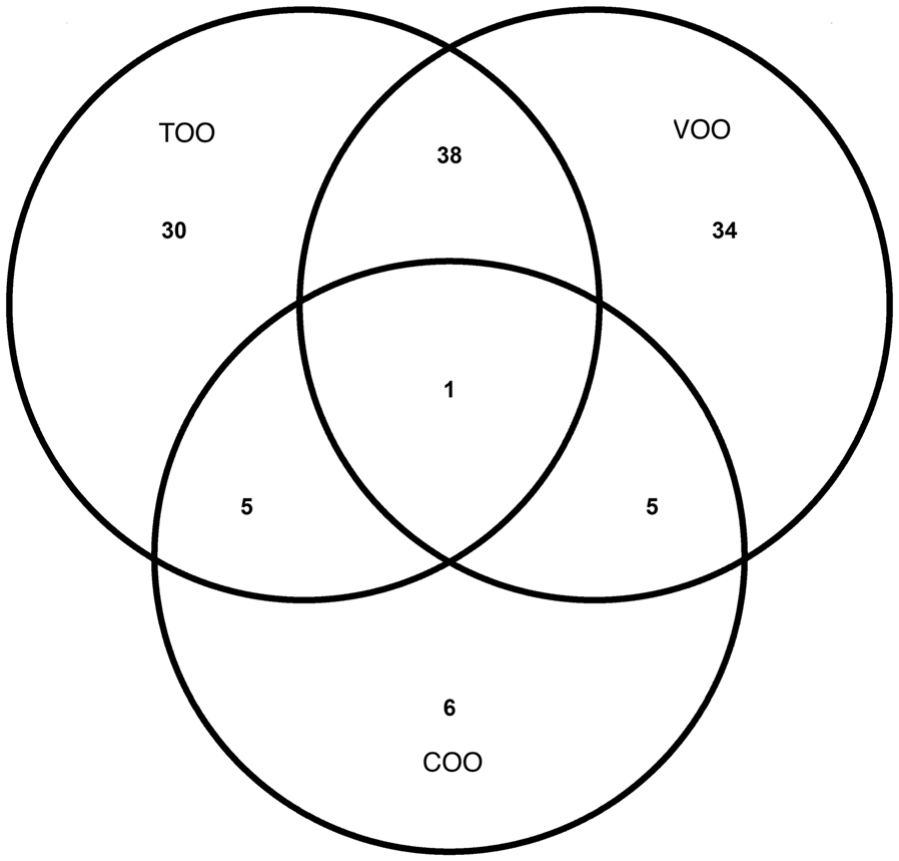
Venn diagram showing the overlapping and different metabolites for total and subtypes of olive oil consumption were identified using elastic net continuous regressions. *TOO* total olive oil, *VOO* virgin olive oil, *COO* common olive oil

Figures 3 and 4 and Additional file 1: Figure S3 show the coefficients (mean and SD) for the ten times selected metabolites in the 10-cross validation of the continuous elastic regression for total olive oil, VOO, and COO. For total olive oil, the metabolites with the strongest inverse associations were C14:2 carnitine, C16:1 cholesterol ester (CE), and cotinine. For VOO, lactose, malate, and sphinganine exhibited the highest negative correlation coefficients; for COO, the strongest inverse associations were found for piperine, pantothenate, and adenosine diphosphate (ADP). For total olive oil, the strongest positive associations were observed for C18:1 carnitine, C34:1 PC plasmalogen A, and C34:2 PC plasmalogen; for VOO, piperine, C34:1 PC plasmalogen A, and ADP showed the strongest direct associations; and for COO, malate, sphingosine, and ornithine were the metabolites with the strongest direct associations. Only piperine was selected in the three metabolomic profiles. This metabolite was positively associated with total olive oil and VOO, but not with COO. Figure 5 shows Venn diagrams showing the number of overlapping and different metabolites for total and subtypes of olive oil identified using elastic net continuous regressions. Thirty metabolites were selected only in the total olive oil profile, thirty-four were only in the VOO profile, and six were only selected in the COO profile.

Furthermore, to identify the principal components consisting of metabolites most associated with total olive oil, VOO, and/or COO, we also performed a PCA using the coefficients of the metabolites selected by the different olive oil consumption profiles with ENR (Additional file 1: Figure S4). The first principal component (1PC) accounted for 53% of the variability, while the second principal component (2PC) accounted for 36.3% of the variability. 1PC differentiated total olive oil and VOO profiles of the COO profile while 2PC showed differences between total olive oil and COO profiles versus VOO profile. In the PCA biplot (Additional file 1: Figure S4), we observed clusters of metabolites clustered close to the three different profiles.

Table 2 shows the Pearson correlation coefficients between each consistently selected metabolite and the consumption of total olive oil, VOO, and COO in the PREDIMED baseline data (discovery population) and 1-year data (validation sample). The Pearson correlations between the metabolite profiles and energy-adjusted olive oil consumption derived from the FFQ at baseline were 0.40 (95% CI: 0.37, 0.44) for total olive oil consumption, 0.37 (95% CI: 0.33, 0.41) for VOO consumption, and 0.25 (95% CI: 0.21, 0.29) for COO consumption. At 1 year, the Pearson correlations were 0.27 (95% CI: 0.22, 0.31) for total olive oil, 0.23 (95% CI: 0.18, 0.28) for VOO, and 0.16 (95% CI: 0.12, 0.21) for COO. Total olive oil consumption was associated with 74 metabolites, VOO with 78, and COO with 17. Additional file 1: Figure S5 shows a correlation plot between the FFQ-derived consumption of total and subtypes of olive oil and metabolite profiles.

**Table 2:** Pearson correlation coefficients between metabolomics signatures and olive oil consumption

	Baseline visit				1-year visit
Assessment	Pearson correlation (95% CI)^1^	Total metabolites^2^	Metabolites with positive coefficients	Metabolites with negative coefficients	Pearson correlation (95% CI)^1^
Total olive oil	0.40 (0.37, 0.44)	74	37	37	0.27 (0.22, 0.31)
Extra virgin olive oil	0.37 (0.33, 0.41)	78	41	37	0.23 (0.18, 0.28)
Common olive oil	0.25 (0.21, 0.29)	17	8	9	0.16 (0.12, 0.21)

^1^The Pearson’s coefficients reflect the correlation between FFQ-derived olive oil consumption and predicted olive oil consumption based on the olive oil-specific multi-metabolite model identified within the discovery cohort

^2^Number of metabolites obtained 10 times in the tenfold cross-validation procedure for the elastic net continuous regression, using the lambda.min option

Olive oil consumption was adjusted for total energy intake by the residual method

### Associations between the identified metabolomic profiles of olive oil consumption and the risk of T2D and CVD

Table 3 shows the prospective associations between the identified metabolomic profiles of total and subtypes of olive oil consumption and the incidence of T2D and CVD. No significant associations were observed for baseline and 1-year olive oil metabolite profiles and T2D risk. The identified baseline metabolomic profiles of total and subtypes of olive oil showed significant associations with CVD incidence. After adjusting for lifestyle and dietary risk factors, the HR for CVD and 95%CI (for every 1 SD increase) was 0.79 (0.67, 0.92; P-value = 0.003) for the total olive oil metabolite profile, and 0.70 (0.59, 0.83; P-value =  < 0.001) for the VOO, but 1.37 (1.15, 1.63; P-value =  < 0.001) for the COO. In sensitivity analysis, additionally adjusting for coffee and tea consumption, the results remained consistent. Only the 1-year VOO metabolite profile was inversely associated with CVD risk (0.81; 0.65, 1.00; P-value = 0.049).

**Table 3:** Hazard ratios (95% CIs) for incident type 2 diabetes and cardiovascular diseases according to multi-metabolite associated with 1 SD of olive oil consumption in the PREDIMED study

Type 2 diabetes				
	Baseline visit^1^		1-year visit^2^	
	HR (95% CI)	*P*	HR (95% CI)	*P*
Cases/total participants	245/923		161/704	
Total olive oil				
Model 1	1.01 (0.85, 1.19)	0.949	1.00 (0.79, 1.27)	0.998
Model 2	1.10 (0.91, 1.32)	0.340	1.04 (0.80, 1.36)	0.765
Model 3	1.11 (0.91, 1.35)	0.296	1.12 (0.84, 1.50)	0.448
Extra virgin olive oil				
Model 1	0.99 (0.82, 1.18)	0.882	0.87 (0.69, 1.09)	0.228
Model 2	1.08 (0.88, 1.33)	0.456	0.90 (0.70, 1.16)	0.415
Model 3	1.08 (0.87, 1.35)	0.479	0.98 (0.74, 1.28)	0.863
Common olive oil				
Model 1	1.01 (0.85, 1.21)	0.882	1.16 (0.89, 1.51)	0.228
Model 2	0.97 (0.79, 1.18)	0.735	1.15 (0.86, 1.54)	0.354
Model 3	0.96 (0.78, 1.19)	0.731	1.06 (0.78, 1.45)	0.700
Cardiovascular disease				
Cases/total participants	222/993		159/916	
Total olive oil				
Model 1	0.77 (0.67, 0.89)	< 0.001	0.90 (0.76, 1.06)	0.194
Model 2	0.77 (0.66, 0.89)	< 0.001	0.89 (0.74, 1.06)	0.178
Model 3	0.79 (0.67, 0.92)	0.003	0.85 (0.69, 1.05)	0.135
Extra virgin olive oil				
Model 1	0.69 (0.59, 0.80)	< 0.001	0.83 (0.70, 0.99)	0.033
Model 2	0.69 (0.58, 0.80)	< 0.001	0.81 (0.67, 0.97)	0.022
Model 3	0.70 (0.59, 0.83)	< 0.001	0.81 (0.65, 1.00)	0.049
Common olive oil				
Model 1	1.41 (1.20, 1.65)	< 0.001	1.18 (0.97, 1.45)	0.099
Model 2	1.39 (1.18, 1.65)	< 0.001	1.17 (0.95, 1.45)	0.136
Model 3	1.37 (1.15, 1.63)	< 0.001	1.17 (0.93, 1.48)	0.184

Model 1: adjusted for age (years), sex, and propensity scores; stratified by intervention group and recruitment center. Model 2: model 1 + BMI, smoking status (never, former, or current smoker), alcohol intake and squared alcohol intake (g/day), education level (primary, secondary, academic) physical activity (metabolic-equivalent minutes per day), family history of CHD (yes/no), dyslipidemia, hypertension, and dyslipidemia and hypertension treatment. Model 3: model 2 + consumption of vegetables, fruits, cereals, nuts, eggs, legumes, fish, meat, and dairy (g/day). Abbreviations: HR, hazard ratio; CI, confidence interval; CVD, cardiovascular disease; T2D, type 2 diabetes; BMI, body mass index

^1^Analysis of T2D risk was conducted in the 923 participants from the PREDIMED-T2D case-cohort database and the analysis of CVD risk was conducted in the 993 participants from the PREDIMED-CVD case-cohort database. Cox proportional hazard models with Barlow weights were used to estimate HRs and their 95% CIs for T2D. Person-time of follow-up was calculated as the interval between the baseline data and the date of T2D or CVD event, death, or date of the last participant contact, whichever came first. HRs refers to a 1-SD increase in correlated multi-metabolite scores

^2^Total olive oil, extra virgin olive oil, and common olive oil metabolic signatures, and covariates were assessed in the first year. The outcome was the incident T2D or CVD events occurred after the first-year visit through to the end of follow-up. The models were the same as in the baseline models. 704 participants for T2D and 916 participants for CVD were included in the analyses

Olive oil consumption variables were adjusted for total energy intake by the residual method

Interactions between olive oil metabolomic profiles and intervention groups were significant in the CVD models (*P*-values < 0.05) and non-significant in the T2D models (*P*-values > 0.05). In the stratified analysis by intervention group (Additional file 1: Tables S2), no significant associations were observed between the olive oil metabolomic profiles and T2D incidence for any of the study intervention groups. However, inverse associations between the metabolite profile of total olive oil and VOO at baseline and the risk of developing CVD were stronger in participants allocated to the MedDiet + VOO group (Additional file 1: Table S3). A direct association between the metabolite signature of COO and a higher risk of CVD was observed. In those participants allocated to the MedDiet + nuts group, only the COO profile showed a positive significant association with CVD incidence. In participants allocated to the control group, an inverse association was observed for the VOO metabolite profile and risk of CVD. At 1 year, only in those participants allocated to the MedDiet + VOO group, a significant inverse association between the total olive oil, and VOO metabolomics signature and CVD was observed, and the COO metabolomic profile was directly associated with CVD risk (Additional file 1: Table S3).

## Discussion

Leveraging the integrated dietary and metabolomics data in a Mediterranean population at high cardiovascular disease risk, using an agnostic machine-learning approach, we identified a metabolomic signature for the consumption of total olive oil (with 74 metabolites), VOO (with 78 metabolites), and COO (with 17 metabolites). The metabolomics signature included several lipids, acylcarnitines, and amino acids. Notably, we identified several overlapping and distinct metabolites according to the type of olive oil consumed. Among the significant findings, certain lipids, including plasmalogens, triacylglycerol (TAG), and the organic acid ADP, exhibited positive associations with total olive oil and virgin olive oil (VOO), while no such correlations were observed with common olive oil (COO). Furthermore, we observed a strong and consistent positive correlation between the multi-metabolite profiles and olive oil consumption as assessed through food-frequency questionnaires (FFQ) at both baseline and one year. Linking these metabolites to disease risk, the metabolite profiles associated with total olive oil and VOO showed inverse associations with CVD risk after adjusting for sociodemographic and dietary factors including FFQ-derived olive oil consumption. However, no significant associations between the multi-metabolite profiles and T2D were observed. To the best of our knowledge, this is the first study evaluating the association between plasma metabolite profiles of total and specific types of olive oil and the risk of cardiometabolic diseases. Overall, our findings provide novel insights into the health-promoting benefits of olive oil consumption and highlight the potential relevance of olive oil-related metabolites in relation to chronic disease risk. These results pave the way for further research on the specific metabolic pathways impacted by olive oil consumption and its implications for preventing cardiometabolic diseases.

Olive oil contains a wide variety of minor phytochemicals, such as tocopherols, carotenoids, and phenolic compounds, with recognized biological activity. Compared to common olive oil (a mixture of refined with a minor quantity of virgin olive oil), VOO is obtained exclusively by physical procedures, such as first-pressing and centrifugation, preserving a large part of its phenolic molecules, mainly hydroxytyrosol, oleocanthal, and oleuropein [30, 31]. Several studies identified a dose-dependent relationship between hydroxytyrosol in plasma or urine and VOO consumption [32, 33]. This metabolite was used as a biomarker of compliance with the intervention (MedDiet + VOO) in the PREDIMED study [12] and its biological metabolite (homovanillyl alcohol) was associated with a lower risk of CVD and total mortality [34]. In the EPIC cohort, urinary hydroxytyrosol was also correlated with total olive oil intake [35]. Unfortunately, our metabolomic platform does not allow us to determine plasma phenolic compounds. In addition, hydroxytyrosol can also be obtained from the endogenous hydroxylation of tyrosol, another common phenolic compound present in beer and wine [36, 37]. The determination of metabolite profiles of total olive oil, VOO, or COO consumption may reflect more specifically the variety of olive oil consumed and the metabolic pathways implicated after its consumption.

In previous analysis within the context of the PREDIMED Study, which evaluated the overall Mediterranean dietary pattern [38], several metabolites selected in the signature were found to be associated with olive oil consumption. Specifically, the consumption of olive oil, measured by the Mediterranean Diet Adherence Score (MEDAS), showed positive correlations with various lipids (C20:5 CE, C22:5 CE, some plasmalogens, and C24:1 SM), C18:1 carnitine and 4-pyridoxate, and negative correlations with other lipid molecules including TAGs, PC, CE or DAG, C4 and C14:2 carnitines, glycine, and pyroglutamic acid. In the present study, we also observed some of the previously mentioned associations in relation to different types of reported olive oil consumption. For instance, C20:5 CE, C18:1 carnitine, and various PCs were positively associated with VOO and/or total olive oil consumption, while C14:2 carnitine or C20:4 LPE were associated negatively. Interestingly, our findings indicate that there was only one metabolite on this list that showed an association with COO consumption. This could be attributed to the relatively low overall consumption of this type of olive oil in our study population when compared to VOO.

While the majority of the metabolites identified in this analysis are involved in internal metabolism, it is noteworthy that some of them originate from food sources or result from microbial activity in the gastrointestinal tract. For instance, piperine showed a positive association with total olive oil and VOO consumption, while exhibiting a negative association with COO consumption. Piperine is an alkaloid found in high concentrations in black pepper [39], which happens to be one of the most commonly used spices in Mediterranean cuisine [40]. Likewise, another interesting finding was the negative association between VOO consumption and ectoine. Ectoine is a secondary metabolite [41, 42] produced by certain bacterial genera, such as Streptomyces spp., and has been identified in small concentrations in the human intestinal microbiota [43]. Its presence in the context of olive oil consumption suggests a potential interaction between the gut microbiome and dietary patterns. Other metabolites derived from intestinal microbiota such as 4-hydroxyhippuric acid, indole-3-propionate, and TMAO were positively related to VOO consumption, and have been associated with the metabolism of polyphenols and the consumption of foods and beverages rich in polyphenols in other studies [44, 45] In previous studies, indole-3-propionate and TMAO were found to have negative associations with VOO and COO consumption, respectively [46]. Additionally, hydroxy cotinine and cotinine (both nicotine derivatives [47], as well as caffeine, AAMU, and N1-metil-2-piridona-5-carboxamide (caffeine derivatives [48, 49]) showed negative associations with total olive oil consumption and/or VOO. These findings may reflect the lower prevalence of smokers and coffee consumers among participants who consume VOO.

Olive oil contains mainly MUFA in the form of oleic acid [50]. Many of the identified metabolites are related to MUFA and lipid metabolic pathways, reflecting the composition of olive oil. Only five lipid metabolites (C14:0 CE, C40:6 PS, C18:2 LPC, C34:5 PC plasmalogen, and C54:5 TAG) were exclusively related to the consumption of COO. C5, C9, and C18:1 carnitines were positively associated with VOO consumption, while C10:2, C12, and C14:2 carnitines were negatively associated. Elevated concentrations of acylcarnitines may be a product of the dysregulation of fatty acid oxidation and mitochondrial function. In the PREDIMED study, medium- and long-chain acylcarnitines have been previously associated with an increased risk of CVD [15], but our results indicated that MedDiet interventions may mitigate the adverse associations shown between higher concentrations of acylcarnitines and CVD.

Among the metabolites related to energy and carbohydrate metabolism, fructose 6-phosphate and inositol were found in the VOO profile, citrate in the total olive oil profile, lactate in both COO and total olive oil profiles, and malate in both VOO and COO profiles. Given that olive oil primarily consists of fatty acids, it is not surprising that only a few carbohydrate-related metabolites were associated with olive oil consumption. Similar observations have been reported in animal studies (51) which showed that diets rich in fats, like the Mediterranean Diet, can influence the Randle cycle, leading to increased malonyl-CoA production from β-oxidation, which serves as a substrate for the TCA cycle and/or gluconeogenesis [52]. Plasma glycocholate, glycodeoxycholic acid, bilirubin, and biliverdin levels are synthesized conjugated bile acids that have been positively associated with total olive oil and/or VOO in our study. Olive oil acts on the gallbladder providing its complete emptying, stimulating the synthesis of bile salts in the liver, and increasing the hepatic secretion of cholesterol [53], thus potentially explaining the association found in our study.

Our signatures have also identified several metabolites related to purine pathways. In our study, 1-methylguanine, urate, indoxyl sulfate (a uremic solute), inosine, adenosine, and ADP were positively associated with VOO or total olive oil consumption. It has been suggested that higher levels of urate in subjects with CVD may represent a compensatory response to counteract oxidative stress [54]. Another oxidative stress marker such as allantoin was negatively associated with VOO but positively associated with common olive oil [55], which suggests that VOO has a key role in these pathways with an antioxidant effect. Some metabolites were identified only in the VOO signature as adipate, acetylcholine, γ-butyrobetaine, 2-aminoadipate, DMGV, pantothenol or trimethyl benzene, and other metabolites only with total olive oil consumption as cytosine or pipecolic acid. We did not find an explanation in the existing literature for why these metabolites showed associations with VOO but not with COO.

These unexplained associations between specific metabolites and VOO or total olive oil consumption could potentially be attributed to the complex interactions between dietary components and individual metabolic responses.

Some metabolites that are part of the identified metabolite profiles have previously been associated with CVD and T2D [56, 57], potentially explaining the positive effects that have been reported for olive oil consumption, especially VOO, on cardiometabolic health [1]. For example, C24:0 ceramide or α-hydroxybutyrate have previously been associated with insulin resistance and increased risk of T2D, and C16:1 CE, several acylcarnitines, cortisol, or deoxycortisol (intermediate of cortisol) with increased CVD risk [56–60]. All these metabolites have been inversely associated with VOO consumption in the signature. However, other lipids such as C54:2 TAG, and C36:2 PC plasmalogen, which have also been positively associated with the consumption of total olive oil or VOO, have been associated with an increased risk of CVD and T2D. After adjusting for potential confounders, we found that the total olive oil and VOO metabolic profiles were associated with a 21% and 26% lower CVD risk, respectively, whereas the COO metabolic profile was associated with a 26% higher risk of CVD risk. However, after 1-year, no statistically significant associations between the total olive oil and COO metabolomic profiles and CVD were observed, similar to the previous analysis of the PREDIMED [61]. In the context of a MedDiet, VOO has been demonstrated to improve lipid profile, markers of glucose and inflammation, and decreased blood pressure, all considered CVD risk factors [1, 62–64]. VOO is known to have a higher concentration of polyphenols compared to COO, and these bioactive compounds are associated with various health benefits, including antioxidant and anti-inflammatory effects or improvements in lipid profile [2]. These observed differences in varying polyphenol content between olive oil subtypes may potentially explain the differences between the metabolomic profiles of VOO and COO and the risk of CVD. Additionally, the statistical differences between VOO and COO profiles could be influenced by the consumption patterns of the study participants, where the lower consumption of COO compared to the consumption of VOO implies an opposite distribution of CVD events. Curiously, similar significant results were seen in the MedDiet groups but not in the control group when stratified by intervention groups. On the other hand, no significant associations were observed between the metabolomic signatures and T2D, and some controversial findings have been reported in the literature for the associations between olive oil consumption and T2D. In the PREDIMED study, while participants allocated to the MedDiet + VOO had a lower risk of developing T2D, the consumption of olive oil alone has not been associated with T2D [65, 66]. Reverse causality might explain these findings. It is possible that participants with higher olive oil consumption, leading to higher T2DM prevalence, had a better overall lifestyle and diet compared to others in the study, influencing the observed outcomes. This highlights the complexity of dietary research and the need for careful consideration of confounding factors.

These findings need to be interpreted in the context of some limitations. First, the identified metabolite profiles are not an objective biomarker of olive oil per se*,* but they reflect the overall homeostasis associated with olive oil consumption, the substitution of other food by the consumption of olive oil, and the individual biological responses to diet. In addition, since olive oil consumption is usually accompanied by other foods, some of the selected metabolites may be associated with the consumption of other foods. Second, the metabolite profiles were derived from a pool of 385 annotated metabolites, while thousands of unique metabolites have been identified to date. We cannot exclude that more biologically relevant metabolites regarding olive oil intake were absent from our data set. For example, the metabolomics approach used for quantifying lipids did not identify the specific fatty acids for each molecule (we can only provide the number of carbons and double bonds of each lipid), and measures of polyphenols and phytochemicals are not available; consequently, some relevant olive oil biomarkers may have been missed. Therefore, the specificity of the identified metabolite profiles of olive oil intake remains uncertain. Future studies are warranted to identify additional objective biomarkers of olive oil intake, including urinary metabolites from dietary intake of phenolic compounds that will be assessed in the future.

Third, due to the use of FFQs for collecting dietary data, measurement errors may be present compared to the use of short-term biomarkers of intake. However, the validity and reproducibility of the FFQ have been reported previously. Of note, the correlation between total olive oil intake assessed by our FFQ and 3-d dietary records was relatively high (r = 0.60) [18]. Because of our study's observational design, we cannot establish the causality of the association between the metabolomic signatures and cardiometabolic diseases. Nevertheless, we performed a rigorous multivariable adjustment to minimize residual confounding. Further, although we evaluated the cross-population reproducibility of the metabolite profiles, it should be validated in independent populations. Our population was a Mediterranean population with a high consumption of VOO even before being randomized in the study. Therefore, we cannot be ruled out reverse causation in participants with diabetes at baseline.

The current study also has several strengths. The PREDIMED study is an ideal setting to identify metabolite profiles associated with olive oil consumption because the consumption of olive oil at baseline (mean total olive oil at baseline in this population is 40 g/d) is much higher than in other populations. In addition, we were able to differentiate between subtypes of olive oil, which have different nutritional compositions, this is a limitation of other existing studies where detailed data on specific subtypes of olive oil and comprehensive metabolomics data are not available. The present study has a large sample size and detailed covariate data to control for confounding and well-defined outcomes. In addition, we used agnostic machine learning models using more than > 350 well-annotated metabolites. We cross-validated our results internally in the discovery population using baseline data and conducted replication analysis using data at year 1.

## Conclusions

In summary, our study analyzed 385 candidate metabolites and identified distinct panels associated with the consumption of total olive oil, VOO, and COO. Specifically, we identified a metabolomic signature associated with the consumption of total olive oil (with 74 metabolites), VOO (with 78 metabolites), and COO (with 17 metabolites). Moreover, our findings highlight the significance of the VOO metabolite profile in reducing the risk of CVD in Mediterranean individuals with high cardiovascular risk. These novel insights provide potential biomarkers of olive oil consumption (especially VOO) and offer valuable information on the mechanisms underlying the relationship between olive oil consumption and cardiometabolic diseases, including CVD and type 2 diabetes (T2D). By advancing our understanding of the metabolic responses to olive oil consumption, our research contributes to the broader field of nutrition and health, emphasizing the health benefits of incorporating extra virgin olive oil into dietary patterns.

## Fundings

The PREDIMED study was funded by NIH grants R01 HL118264, R01 DK102896 and R01 3R01 HL118264, the Spanish Ministry of Health (Instituto de Salud Carlos III, The PREDIMED Network grant RD 06/0045, 2006–2013, coordinated by M.A. Martínez-González; and a previous network grant RTIC-G03/140, 2003–2005, coordinated R. Estruch). Additional grants were received from the Ministerio de Economía y Competitividad-Fondo Europeo de Desarrollo Regional (Projects CNIC-06/2007, CIBER 06/03, PI06-1326, PI07-0954, PI11/02505, SAF2009-12304, and AGL2010–22319-C03-03) and by the Generalitat Valenciana (PROMETEO17/2017 and PROMETEO 21/2021). Dr. Guasch-Ferré acknowledges that The Novo Nordisk Foundation Center for Basic Metabolic Research is supported by and unrestricted grant from the Novo Nordisk Foundation (grant no. NNF18CC0034900). Dr. Salas-Salvadó gratefully acknowledges the financial support by ICREA under the ICREA Academia program.

### Supplementary Information

**Additional file1: Table S1.** Associations between plasma metabolites and total olive oil, extra virgin olive oil, and common olive oil consumption at baseline. **Table S2.** Hazard ratios (95% CIs) of type 2 diabetes incidence according to metabolomics profiles of energy-adjusted olive oil and its subtypes in the PREDIMED study groups stratified by intervention group. **Table S3.** Hazard ratios (95% CIs) of cardiovascular disease incidence according to metabolomics profiles of energy-adjusted olive oil and its subtypes in the PREDIMED study groups stratified by intervention group. **Figure S1.** Flowchart of participants and analysis. **Figure S2.** Volcano plot showing the associations between plasma metabolites and common olive oil consumption at baseline. **Figure S3.** Metabolite coefficients (mean and SD) selected ten times in the 10-cross validation of the continuous elastic regression for energy-adjusted common olive oil. **Figure S4.** Biplot of the principal component analysis using metabolites’ coefficients derived by the elastic net continuous regression of each olive oil consumption approach. **Figure S5.** Correlation plot showing the correlation between each self-reported olive oil intake and olive oil metabolomics profiles and each food group consumption.

## Data Availability

The dataset generated and/or analyzed during the current study is not publicly available due to the lack of authorization from PREDIMED participants. Those wishing to access the PREDIMED trial data used in this study can request the corresponding author and it will then be passed on to members of the PREDIMED Steering Committee for deliberation.

## Abbreviations

95% CI: 95% Confidence interval
AAMU: 5-Acetamido-6-amino-3-methyl-uracil
ADP: Adenosine diphosphate
BMI: Body mass index
CE: Cholesterol ester
COO: Common olive oil
CV: Cross-validation
CVD: Cardiovascular diseases
DAG: Diacylglycerol
DMGV: Dimethylguanidino valeric acid
VOO: Virgin olive oil
FFQ: Food frequency questionnaires
HR: Hazard ratio
LC-MS: Liquid chromatography-tandem mass spectrometry
LPC: Lysophosphatidylcholine
LPE: Lysophosphatidylethanolamine
MAG: Monoacylglycerol
MEDAS: Mediterranean Diet Adherence Screener
MedDiet: Mediterranean Diet
MUFA: Monounsaturated fatty acid
PC: Phosphatidylcholine
PREDIMED: PREvención con DIeta MEDiterránea
PS: Phosphatidylserine
SM: Sphingomyelin
T2D: Type 2 diabetes
TAG: Triacylglycerol
TCA: Tricarboxylic acid cycle
TMAO: Trimethylamine N-oxide
