# Supplementary material for: Olive oil consumption, plasma metabolites, and risk of type 2 diabetes and cardiovascular disease
Source: Cardiovasc Diabetol. 2023 Dec 13;22:340. doi: 10.1186/s12933-023-02066-1 (PMC10720204; doi:10.1186/s12933-023-02066-1)

[**Additional Table 1.** Associations between plasma metabolites and total olive oil, extra virgin olive oil, and common olive oil consumption at baseline. 2](#_Toc140678388)

[**Additional Table 2**. Hazard ratios (95% CIs) of type 2 diabetes incidence according to metabolomics profiles of energy-adjusted olive oil and its subtypes in the PREDIMED study groups stratified by intervention group. 17](#_Toc140678389)

[**Additional Table 3**. Hazard ratios (95% CIs) of cardiovascular disease incidence according to metabolomics profiles of energy-adjusted olive oil and its subtypes in the PREDIMED study groups stratified by intervention group. 18](#_Toc140678390)

[**Additional Figure 1**. Flowchart of participants and analysis. 19](#_Toc140678391)

[**Additional Figure 2**. Volcano plot showing the associations between plasma metabolites and common olive oil consumption at baseline. 20](#_Toc140678392)

[**Additional Figure 3**. Metabolite coefficients (mean and SD) selected ten times in the 10-cross validation of the continuous elastic regression for energy-adjusted common olive oil. 21](#_Toc140678393)

[**Additional Figure 4**. Biplot of the principal component analysis using metabolites’ coefficients derived by the elastic net continuous regression of each olive oil consumption approach. 22](#_Toc140678394)

[**Additional Figure 5**. Correlation plot showing the correlation between each self-reported olive oil intake and olive oil metabolomics profiles and each food group consumption. 23](#_Toc140678395)

**Additional Table 1.** Associations between plasma metabolites and total olive oil, extra virgin olive oil, and common olive oil consumption at baseline.

|  | **Total Olive oil** |  | **Extra virgin olive oil** |  | **Common olive oil** | |
| --- | --- | --- | --- | --- | --- | --- |
| **Metabolite** | **Β Coefficient (95% IC)** | ***P*-value** | **Β Coefficient (95% IC)** | ***P*-value** | **Β Coefficient (95% IC)** | ***P*-value** |
| 1-Methyladenosine | -0.003 (-0.005; 0.000) | 0.080 | -0.002 (-0.004; 0.000) | 0.047 | 0.001 (-0.001; 0.003) | 0.411 |
| 1-Methylguanine | 0.002 (-0.001; 0.005) | 0.175 | 0.000 (-0.002; 0.002) | 0.688 | 0.002 (-0.001; 0.004) | 0.138 |
| 1-Methylguanosine | 0.000 (-0.003; 0.003) | 0.819 | 0.001 (-0.001; 0.003) | 0.484 | -0.001 (-0.003; 0.001) | 0.417 |
| 1-Methylhistamine | -0.003 (-0.006; 0.000) | 0.037 | -0.001 (-0.003; 0.001) | 0.279 | -0.001 (-0.003; 0.002) | 0.566 |
| 1-Methylhistidine | 0.003 (0.000; 0.006) | 0.062 | 0.001 (-0.001; 0.003) | 0.465 | 0.001 (-0.002; 0.003) | 0.536 |
| 1-methylnicotinamide | 0.001 (-0.002; 0.004) | 0.473 | 0.001 (-0.001; 0.003) | 0.426 | 0.000 (-0.003; 0.002) | 0.706 |
| 1-methylnicotinamide | 0.001 (-0.002; 0.004) | 0.526 | 0.001 (-0.001; 0.003) | 0.590 | 0.000 (-0.003; 0.002) | 0.867 |
| 2-aminoadipate | 0.002 (-0.001; 0.004) | 0.288 | 0.002 (0.000; 0.004) | 0.108 | -0.001 (-0.003; 0.001) | 0.363 |
| 2-Hydroxyglutarate | -0.002 (-0.005; 0.001) | 0.214 | -0.001 (-0.003; 0.001) | 0.523 | 0.000 (-0.003; 0.002) | 0.840 |
| 3-Hydroxyanthranilic acid | 0.001 (-0.002; 0.004) | 0.385 | 0.000 (-0.002; 0.002) | 0.864 | 0.001 (-0.002; 0.003) | 0.495 |
| 3-Methyladipate-pimelate | 0.000 (-0.003; 0.003) | 0.869 | 0.000 (-0.002; 0.002) | 0.945 | 0.000 (-0.002; 0.003) | 0.756 |
| 4-Acetamidobutanoate | -0.002 (-0.005; 0.001) | 0.175 | -0.001 (-0.003; 0.001) | 0.526 | 0.000 (-0.003; 0.002) | 0.747 |
| 4-Guanidinobutanoic acid | -0.001 (-0.004; 0.002) | 0.468 | 0.000 (-0.002; 0.002) | 0.755 | -0.001 (-0.003; 0.001) | 0.425 |
| 4-Hydroxy-3-methylacetophenone | -0.001 (-0.004; 0.001) | 0.314 | 0.001 (-0.001; 0.003) | 0.509 | -0.002 (-0.004; 0.001) | 0.133 |
| 4-Hydroxyhippurate | 0.002 (-0.001; 0.005) | 0.109 | 0.001 (-0.001; 0.004) | 0.157 | 0.000 (-0.003; 0.002) | 0.904 |
| 4-Pyridoxate | 0.001 (-0.002; 0.004) | 0.543 | 0.001 (-0.001; 0.003) | 0.315 | -0.001 (-0.003; 0.002) | 0.481 |
| 7-methylguanine | -0.001 (-0.004; 0.002) | 0.452 | -0.002 (-0.004; 0.000) | 0.114 | 0.002 (-0.001; 0.004) | 0.164 |
| AAMU | -0.003 (-0.006; 0.000) | 0.042 | -0.002 (-0.004; 0.000) | 0.035 | 0.001 (-0.001; 0.003) | 0.379 |
| Acetylcholine | -0.001 (-0.004; 0.002) | 0.583 | -0.002 (-0.004; 0.000) | 0.128 | 0.002 (-0.001; 0.004) | 0.178 |
| Aconitate | -0.001 (-0.004; 0.002) | 0.404 | -0.001 (-0.003; 0.001) | 0.438 | 0.001 (-0.002; 0.003) | 0.609 |
| Adenosine | -0.002 (-0.005; 0.001) | 0.205 | 0.001 (-0.001; 0.003) | 0.546 | -0.002 (-0.004; 0.001) | 0.134 |
| Adipate | 0.000 (-0.003; 0.003) | 0.807 | -0.001 (-0.003; 0.001) | 0.390 | 0.001 (-0.001; 0.004) | 0.344 |
| ADMA | 0.003 (0.000; 0.006) | 0.042 | 0.001 (-0.001; 0.003) | 0.252 | 0.001 (-0.001; 0.003) | 0.391 |
| ADP | -0.002 (-0.005; 0.001) | 0.178 | 0.001 (-0.001; 0.003) | 0.476 | -0.003 (-0.005; 0.000) | 0.025 |
| Alanine | 0.000 (-0.003; 0.003) | 0.970 | -0.002 (-0.004; 0.000) | 0.116 | 0.002 (0.000; 0.005) | 0.068 |
| Allantoin | 0.001 (-0.002; 0.004) | 0.603 | -0.001 (-0.003; 0.001) | 0.231 | 0.002 (0.000; 0.005) | 0.049 |
| Alpha-aminoisobutyric acid | 0.002 (-0.001; 0.005) | 0.111 | 0.001 (-0.001; 0.003) | 0.372 | 0.000 (-0.002; 0.002) | 0.992 |
| alpha-glycerophosphate | 0.001 (-0.002; 0.004) | 0.482 | 0.000 (-0.002; 0.002) | 0.983 | 0.001 (-0.001; 0.003) | 0.406 |
| Alpha-GPC | -0.003 (-0.006; -0.001) | 0.019 | -0.002 (-0.004; 0.000) | 0.055 | 0.000 (-0.002; 0.003) | 0.717 |
| Alpha-hydroxybutyrate | 0.004 (0.002; 0.007) | **0.002** | 0.002 (0.000; 0.004) | 0.018 | 0.000 (-0.002; 0.002) | 0.857 |
| AMP | -0.004 (-0.007; -0.001) | **0.003** | -0.002 (-0.004; 0.000) | 0.100 | -0.001 (-0.003; 0.002) | 0.491 |
| Arginine | -0.001 (-0.003; 0.002) | 0.708 | 0.000 (-0.002; 0.002) | 0.764 | 0.000 (-0.003; 0.002) | 0.743 |
| Asparagine | 0.002 (-0.001; 0.004) | 0.272 | -0.001 (-0.003; 0.001) | 0.569 | 0.002 (0.000; 0.005) | 0.073 |
| Beta-alanine | 0.001 (-0.002; 0.004) | 0.666 | 0.000 (-0.002; 0.002) | 0.964 | 0.001 (-0.002; 0.003) | 0.657 |
| Beta-hydroxybutyrate | 0.000 (-0.003; 0.003) | 0.916 | 0.002 (0.000; 0.004) | 0.106 | -0.002 (-0.004; 0.001) | 0.151 |
| Betaine | 0.003 (0.000; 0.006) | 0.041 | 0.002 (0.000; 0.004) | 0.108 | 0.000 (-0.002; 0.002) | 0.881 |
| Bilirubin | 0.004 (0.002; 0.007) | **0.002** | 0.003 (0.001; 0.005) | 0.003 | -0.001 (-0.003; 0.001) | 0.434 |
| Biliverdin | 0.004 (0.001; 0.007) | 0.008 | 0.002 (0.000; 0.004) | 0.049 | 0.000 (-0.002; 0.002) | 0.888 |
| C10 carnitine | -0.001 (-0.004; 0.002) | 0.497 | 0.000 (-0.002; 0.002) | 0.979 | -0.001 (-0.004; 0.001) | 0.317 |
| C10:2 carnitine | -0.003 (-0.006; 0.000) | 0.042 | -0.001 (-0.003; 0.001) | 0.344 | -0.001 (-0.003; 0.001) | 0.440 |
| C12 carnitine | -0.003 (-0.005; 0.000) | 0.083 | -0.001 (-0.004; 0.001) | 0.165 | 0.000 (-0.002; 0.002) | 0.997 |
| C12:1 carnitine | 0.000 (-0.003; 0.003) | 0.868 | 0.000 (-0.002; 0.002) | 0.908 | 0.000 (-0.003; 0.002) | 0.902 |
| C14 carnitine | -0.003 (-0.006; 0.000) | 0.068 | -0.002 (-0.005; 0.000) | 0.020 | 0.002 (-0.001; 0.004) | 0.212 |
| C14:0 CE | 0.001 (-0.002; 0.004) | 0.365 | -0.002 (-0.004; 0.000) | 0.070 | 0.004 (0.001; 0.006) | 0.004 |
| C14:0 LPC | -0.003 (-0.006; 0.000) | 0.042 | -0.003 (-0.005; -0.001) | **0.002** | 0.003 (0.000; 0.005) | 0.035 |
| C14:0 SM | -0.002 (-0.004; 0.001) | 0.193 | -0.001 (-0.003; 0.001) | 0.168 | 0.001 (-0.001; 0.003) | 0.515 |
| C14:1 carnitine | 0.002 (-0.001; 0.005) | 0.194 | 0.001 (-0.001; 0.003) | 0.382 | 0.000 (-0.002; 0.002) | 0.966 |
| C14:1 MAG | 0.002 (-0.001; 0.005) | 0.111 | 0.002 (0.000; 0.004) | 0.136 | 0.000 (-0.003; 0.002) | 0.736 |
| C14:2 carnitine | -0.008 (-0.010; -0.005) | **<0.001** | -0.002 (-0.004; 0.000) | 0.056 | -0.003 (-0.005; 0.000) | 0.034 |
| C16 carnitine | 0.000 (-0.003; 0.003) | 0.856 | 0.000 (-0.002; 0.002) | 0.958 | 0.000 (-0.002; 0.003) | 0.718 |
| C16:0 CE | 0.000 (-0.002; 0.003) | 0.811 | 0.001 (-0.001; 0.003) | 0.601 | -0.001 (-0.003; 0.002) | 0.569 |
| C16:0 Ceramide d18:1 | 0.001 (-0.002; 0.004) | 0.647 | 0.000 (-0.002; 0.002) | 0.867 | 0.000 (-0.003; 0.002) | 0.878 |
| C16:0 LPC | -0.001 (-0.004; 0.002) | 0.366 | -0.001 (-0.003; 0.001) | 0.500 | 0.000 (-0.002; 0.002) | 0.999 |
| C16:0 LPE | -0.002 (-0.005; 0.000) | 0.103 | -0.001 (-0.003; 0.001) | 0.270 | 0.000 (-0.003; 0.002) | 0.901 |
| C16:0 SM | 0.003 (0.000; 0.006) | 0.030 | 0.001 (-0.001; 0.003) | 0.158 | 0.000 (-0.002; 0.002) | 0.935 |
| C16:1 CE | 0.000 (-0.003; 0.003) | 0.879 | -0.002 (-0.004; 0.000) | 0.107 | 0.003 (0.000; 0.005) | 0.031 |
| C16:1 LPC | -0.001 (-0.004; 0.001) | 0.308 | -0.002 (-0.004; 0.000) | 0.068 | 0.002 (-0.001; 0.004) | 0.138 |
| C16:1 MAG | 0.002 (-0.001; 0.004) | 0.305 | 0.002 (0.000; 0.004) | 0.124 | -0.001 (-0.004; 0.001) | 0.326 |
| C16:1 SM | 0.001 (-0.001; 0.004) | 0.319 | 0.001 (-0.001; 0.003) | 0.316 | -0.001 (-0.003; 0.001) | 0.573 |
| C18 carnitine | -0.001 (-0.004; 0.001) | 0.312 | -0.001 (-0.003; 0.002) | 0.617 | 0.000 (-0.003; 0.002) | 0.750 |
| C18:0 CE | 0.001 (-0.002; 0.004) | 0.443 | -0.001 (-0.002; 0.001) | 0.615 | 0.001 (-0.001; 0.003) | 0.408 |
| C18:0 LPC | -0.002 (-0.005; 0.001) | 0.198 | -0.001 (-0.003; 0.001) | 0.289 | 0.000 (-0.002; 0.002) | 0.940 |
| C18:0 LPE | -0.005 (-0.008; -0.002) | **0.001** | -0.002 (-0.004; 0.000) | 0.074 | -0.001 (-0.003; 0.002) | 0.531 |
| C18:0 MAG | 0.004 (0.002; 0.007) | **0.002** | 0.002 (-0.001; 0.004) | 0.143 | 0.001 (-0.001; 0.003) | 0.387 |
| C18:0 SM | 0.003 (0.001; 0.006) | **0.017** | 0.001 (-0.001; 0.003) | 0.255 | 0.000 (-0.002; 0.002) | 0.851 |
| C18:1 carnitine | 0.010 (0.007; 0.013) | **<0.001** | 0.003 (0.001; 0.005) | 0.001 | 0.003 (0.000; 0.005) | 0.022 |
| C18:1 CE | 0.008 (0.005; 0.011) | **<0.001** | 0.003 (0.001; 0.005) | **0.004** | 0.001 (-0.001; 0.004) | 0.271 |
| C18:1 LPC | 0.006 (0.003; 0.009) | **<0.001** | 0.002 (0.000; 0.004) | 0.019 | 0.001 (-0.002; 0.003) | 0.574 |
| C18:1 LPE | 0.002 (-0.001; 0.005) | 0.129 | 0.002 (0.000; 0.004) | 0.084 | -0.001 (-0.003; 0.002) | 0.540 |
| C18:1 SM | 0.003 (0.001; 0.006) | **0.008** | 0.002 (0.000; 0.003) | 0.060 | -0.001 (-0.003; 0.001) | 0.551 |
| C18:1-OH carnitine | 0.004 (0.001; 0.006) | 0.012 | 0.001 (-0.001; 0.003) | 0.292 | 0.001 (-0.001; 0.004) | 0.314 |
| C18:2 carnitine | -0.003 (-0.006; 0.000) | 0.036 | -0.002 (-0.004; 0.000) | 0.116 | 0.000 (-0.002; 0.003) | 0.838 |
| C18:2 CE | 0.001 (-0.002; 0.004) | 0.429 | 0.001 (-0.001; 0.003) | 0.406 | -0.001 (-0.003; 0.002) | 0.617 |
| C18:2 LPC | -0.004 (-0.007; -0.001) | **0.008** | 0.000 (-0.002; 0.002) | 0.800 | -0.002 (-0.005; 0.000) | 0.064 |
| C18:2 LPE | -0.005 (-0.008; -0.003) | **<0.001** | -0.001 (-0.004; 0.001) | 0.169 | -0.002 (-0.004; 0.001) | 0.204 |
| C18:2 SM | 0.002 (0.000; 0.005) | 0.065 | 0.001 (-0.001; 0.003) | 0.162 | 0.000 (-0.003; 0.002) | 0.702 |
| C18:3 CE | 0.001 (-0.002; 0.003) | 0.642 | -0.001 (-0.003; 0.001) | 0.290 | 0.002 (0.000; 0.004) | 0.069 |
| C2 carnitine | 0.001 (-0.002; 0.004) | 0.496 | 0.000 (-0.002; 0.002) | 0.959 | 0.001 (-0.001; 0.003) | 0.400 |
| C20 carnitine | 0.001 (-0.002; 0.004) | 0.616 | 0.000 (-0.002; 0.002) | 0.680 | 0.001 (-0.001; 0.003) | 0.399 |
| C20:0 LPE | -0.001 (-0.004; 0.002) | 0.397 | 0.000 (-0.002; 0.002) | 0.737 | -0.001 (-0.003; 0.002) | 0.605 |
| C20:0 SM | 0.002 (-0.001; 0.005) | 0.138 | 0.001 (-0.001; 0.003) | 0.248 | 0.000 (-0.003; 0.002) | 0.757 |
| C20:3 CE | 0.003 (0.000; 0.005) | 0.070 | 0.000 (-0.002; 0.002) | 0.759 | 0.002 (0.000; 0.004) | 0.069 |
| C20:3 LPC | -0.001 (-0.004; 0.002) | 0.377 | -0.001 (-0.003; 0.001) | 0.530 | 0.000 (-0.002; 0.002) | 0.962 |
| C20:4 carnitine | 0.003 (0.000; 0.005) | 0.074 | 0.000 (-0.002; 0.002) | 0.764 | 0.002 (-0.001; 0.004) | 0.135 |
| C20:4 CE | 0.003 (0.000; 0.006) | 0.025 | 0.001 (-0.001; 0.003) | 0.168 | 0.000 (-0.002; 0.002) | 0.947 |
| C20:4 LPC | -0.004 (-0.007; -0.001) | **0.012** | -0.001 (-0.003; 0.001) | 0.228 | -0.001 (-0.003; 0.002) | 0.521 |
| C20:4 LPE | -0.005 (-0.008; -0.002) | **0.002** | -0.001 (-0.004; 0.001) | 0.166 | -0.001 (-0.003; 0.001) | 0.427 |
| C20:5 CE | 0.008 (0.005; 0.010) | **<0.001** | 0.003 (0.001; 0.005) | 0.011 | 0.001 (-0.001; 0.004) | 0.245 |
| C20:5 LPC | 0.002 (-0.001; 0.004) | 0.260 | 0.001 (-0.001; 0.003) | 0.535 | 0.000 (-0.002; 0.003) | 0.825 |
| C22:0 Ceramide d18:1 | -0.002 (-0.005; 0.001) | 0.181 | -0.001 (-0.003; 0.002) | 0.593 | -0.001 (-0.003; 0.002) | 0.549 |
| C22:0 LPE | 0.002 (-0.001; 0.004) | 0.276 | 0.001 (-0.001; 0.003) | 0.263 | -0.001 (-0.003; 0.002) | 0.668 |
| C22:0 SM | -0.001 (-0.004; 0.002) | 0.410 | -0.001 (-0.003; 0.001) | 0.239 | 0.001 (-0.002; 0.003) | 0.615 |
| C22:1 MAG | 0.003 (0.000; 0.006) | 0.038 | 0.001 (-0.001; 0.003) | 0.355 | 0.001 (-0.002; 0.003) | 0.592 |
| C22:1 SM | 0.001 (-0.001; 0.003) | 0.425 | 0.000 (-0.002; 0.002) | 0.822 | 0.000 (-0.002; 0.002) | 0.893 |
| C22:4 CE | -0.004 (-0.007; -0.001) | **0.012** | -0.001 (-0.003; 0.001) | 0.181 | -0.001 (-0.003; 0.002) | 0.609 |
| C22:5 CE | 0.003 (0.000; 0.006) | 0.060 | 0.001 (-0.001; 0.003) | 0.291 | 0.000 (-0.002; 0.002) | 0.979 |
| C22:6 CE | 0.008 (0.005; 0.010) | **<0.001** | 0.004 (0.002; 0.006) | **<0.001** | 0.000 (-0.002; 0.002) | 0.927 |
| C22:6 LPC | 0.002 (-0.001; 0.004) | 0.298 | 0.001 (-0.001; 0.003) | 0.434 | 0.000 (-0.003; 0.002) | 0.918 |
| C22:6 LPE | 0.000 (-0.003; 0.003) | 0.846 | 0.001 (-0.001; 0.003) | 0.572 | 0.000 (-0.003; 0.002) | 0.694 |
| C24:0 Ceramide d18:1 | -0.002 (-0.005; 0.000) | 0.097 | -0.001 (-0.003; 0.001) | 0.320 | 0.000 (-0.003; 0.002) | 0.706 |
| C24:0 SM | -0.002 (-0.005; 0.001) | 0.119 | -0.002 (-0.004; 0.000) | 0.079 | 0.001 (-0.002; 0.003) | 0.597 |
| C24:1 Ceramide d18:1 | 0.008 (0.005; 0.011) | **<0.001** | 0.004 (0.002; 0.006) | **<0.001** | 0.000 (-0.002; 0.002) | 0.961 |
| C24:1 SM | 0.007 (0.005; 0.010) | **<0.001** | 0.003 (0.001; 0.005) | **0.004** | 0.001 (-0.002; 0.003) | 0.545 |
| C26 carnitine | 0.001 (-0.002; 0.004) | 0.498 | 0.001 (-0.001; 0.003) | 0.365 | 0.000 (-0.003; 0.002) | 0.725 |
| C3 carnitine | -0.001 (-0.004; 0.002) | 0.605 | 0.000 (-0.002; 0.002) | 0.994 | 0.000 (-0.003; 0.002) | 0.759 |
| C30:0 DAG | -0.001 (-0.003; 0.002) | 0.673 | -0.001 (-0.003; 0.001) | 0.489 | 0.001 (-0.002; 0.003) | 0.520 |
| C30:0 PC | -0.003 (-0.006; 0.000) | 0.064 | -0.003 (-0.005; -0.001) | **0.002** | 0.003 (0.000; 0.005) | 0.028 |
| C30:1 PC | -0.002 (-0.005; 0.001) | 0.150 | -0.003 (-0.005; -0.001) | **0.001** | 0.003 (0.001; 0.006) | 0.004 |
| C32:0 DAG | -0.003 (-0.005; 0.000) | 0.076 | -0.001 (-0.003; 0.001) | 0.275 | 0.000 (-0.002; 0.002) | 0.986 |
| C32:0 PC | 0.001 (-0.002; 0.004) | 0.424 | 0.000 (-0.002; 0.002) | 0.791 | 0.000 (-0.002; 0.003) | 0.736 |
| C32:0 PE | -0.003 (-0.005; 0.000) | 0.075 | -0.003 (-0.006; -0.001) | **<0.001** | 0.003 (0.001; 0.005) | 0.010 |
| C32:1 DAG | -0.002 (-0.005; 0.000) | 0.087 | -0.002 (-0.004; 0.000) | 0.066 | 0.001 (-0.001; 0.004) | 0.333 |
| C32:1 PC | -0.001 (-0.004; 0.002) | 0.600 | -0.002 (-0.004; 0.000) | 0.060 | 0.002 (0.000; 0.005) | 0.056 |
| C32:2 PC | -0.006 (-0.009; -0.003) | **<0.001** | -0.004 (-0.006; -0.002) | **<0.001** | 0.001 (-0.001; 0.003) | 0.317 |
| C34:0 DAG | 0.003 (0.000; 0.006) | 0.041 | 0.001 (-0.001; 0.003) | 0.241 | 0.000 (-0.002; 0.003) | 0.766 |
| C34:0 PC | 0.000 (-0.003; 0.003) | 0.825 | 0.000 (-0.002; 0.002) | 0.804 | 0.000 (-0.003; 0.002) | 0.722 |
| C34:0 PE | -0.002 (-0.005; 0.001) | 0.120 | -0.003 (-0.005; -0.001) | **0.004** | 0.002 (0.000; 0.005) | 0.035 |
| C34:0 PI | -0.002 (-0.005; 0.001) | 0.207 | -0.001 (-0.003; 0.001) | 0.293 | 0.000 (-0.002; 0.002) | 0.959 |
| C34:0 PS | 0.006 (0.003; 0.009) | **<0.001** | 0.002 (0.000; 0.004) | 0.077 | 0.001 (-0.001; 0.004) | 0.217 |
| C34:1 DAG | 0.001 (-0.002; 0.004) | 0.522 | 0.000 (-0.002; 0.002) | 0.880 | 0.001 (-0.002; 0.003) | 0.652 |
| C34:1 PC | 0.007 (0.004; 0.010) | **<0.001** | 0.002 (0.000; 0.004) | 0.092 | 0.002 (0.000; 0.005) | 0.043 |
| C34:1 PC plasmalogenA | 0.010 (0.008; 0.013) | **<0.001** | 0.005 (0.003; 0.007) | **<0.001** | 0.001 (-0.001; 0.003) | 0.446 |
| C34:1 PC plasmalogenB | 0.002 (-0.001; 0.005) | 0.157 | 0.001 (-0.001; 0.003) | 0.597 | 0.000 (-0.002; 0.003) | 0.699 |
| C34:2 DAG | -0.004 (-0.006; -0.001) | **0.014** | -0.001 (-0.003; 0.001) | 0.198 | 0.000 (-0.003; 0.002) | 0.699 |
| C34:2 hydroxy-PC | 0.000 (-0.003; 0.002) | 0.801 | 0.001 (-0.001; 0.003) | 0.447 | -0.002 (-0.004; 0.001) | 0.164 |
| C34:2 PC | -0.003 (-0.006; 0.000) | 0.057 | -0.001 (-0.003; 0.001) | 0.591 | -0.001 (-0.003; 0.001) | 0.360 |
| C34:2 PC plasmalogen | 0.009 (0.007; 0.012) | **<0.001** | 0.004 (0.002; 0.006) | **<0.001** | 0.001 (-0.001; 0.003) | 0.462 |
| C34:2 PE | -0.005 (-0.007; -0.002) | **0.001** | -0.002 (-0.004; 0.000) | 0.076 | 0.000 (-0.003; 0.002) | 0.690 |
| C34:2 PE plasmalogen | 0.006 (0.003; 0.009) | **<0.001** | 0.003 (0.001; 0.005) | **0.002** | 0.000 (-0.003; 0.002) | 0.778 |
| C34:3 DAG | -0.007 (-0.010; -0.004) | **<0.001** | -0.002 (-0.005; 0.000) | 0.017 | -0.001 (-0.004; 0.001) | 0.309 |
| C34:3 PC | -0.003 (-0.006; -0.001) | 0.015 | -0.002 (-0.004; 0.000) | 0.014 | 0.001 (-0.001; 0.003) | 0.310 |
| C34:3 PC plasmalogen | -0.003 (-0.005; 0.000) | 0.067 | -0.001 (-0.003; 0.001) | 0.539 | -0.001 (-0.004; 0.001) | 0.245 |
| C34:3 PE plasmalogen | -0.002 (-0.005; 0.000) | 0.093 | 0.000 (-0.002; 0.002) | 0.673 | -0.001 (-0.004; 0.001) | 0.291 |
| C34:4 PC | -0.005 (-0.007; -0.002) | **0.001** | -0.003 (-0.005; -0.001) | **0.001** | 0.002 (-0.001; 0.004) | 0.193 |
| C34:5 PC plasmalogen | 0.000 (-0.003; 0.003) | 0.817 | 0.002 (0.000; 0.004) | 0.051 | -0.003 (-0.005; 0.000) | 0.024 |
| C36:0 DAG | 0.003 (0.000; 0.006) | 0.026 | 0.002 (0.000; 0.004) | 0.047 | -0.001 (-0.003; 0.002) | 0.529 |
| C36:0 PC | 0.000 (-0.002; 0.003) | 0.757 | 0.000 (-0.002; 0.002) | 0.831 | 0.000 (-0.003; 0.002) | 0.792 |
| C36:1 DAG | 0.001 (-0.002; 0.003) | 0.698 | 0.000 (-0.002; 0.002) | 0.936 | 0.000 (-0.002; 0.003) | 0.808 |
| C36:1 PC | 0.008 (0.005; 0.011) | **<0.001** | 0.002 (0.000; 0.004) | 0.096 | 0.003 (0.001; 0.005) | 0.012 |
| C36:1 PC plasmalogen | 0.007 (0.004; 0.010) | **<0.001** | 0.002 (0.000; 0.004) | 0.042 | 0.002 (-0.001; 0.004) | 0.163 |
| C36:1 PE | 0.002 (-0.001; 0.005) | 0.120 | 0.001 (-0.001; 0.003) | 0.178 | 0.000 (-0.003; 0.002) | 0.791 |
| C36:1 PE plasmalogen | 0.001 (-0.002; 0.004) | 0.569 | 0.001 (-0.002; 0.003) | 0.608 | 0.000 (-0.002; 0.002) | 0.950 |
| C36:1 PS plasmalogen | 0.007 (0.004; 0.010) | **<0.001** | 0.003 (0.001; 0.005) | 0.014 | 0.001 (-0.001; 0.004) | 0.304 |
| C36:2 DAG | 0.006 (0.003; 0.008) | **<0.001** | 0.002 (0.000; 0.004) | 0.023 | 0.001 (-0.002; 0.003) | 0.616 |
| C36:2 PC | 0.001 (-0.002; 0.003) | 0.687 | 0.000 (-0.002; 0.002) | 0.805 | 0.000 (-0.002; 0.002) | 0.996 |
| C36:2 PC plasmalogen | 0.007 (0.005; 0.010) | **<0.001** | 0.003 (0.001; 0.005) | **<0.001** | 0.000 (-0.002; 0.003) | 0.855 |
| C36:2 PE | -0.003 (-0.006; 0.000) | 0.037 | -0.001 (-0.003; 0.001) | 0.576 | -0.001 (-0.004; 0.001) | 0.332 |
| C36:2 PE plasmalogen | 0.005 (0.002; 0.008) | **<0.001** | 0.003 (0.001; 0.005) | **0.006** | -0.001 (-0.003; 0.002) | 0.555 |
| C36:2 PS plasmalogen | -0.003 (-0.006; 0.000) | 0.031 | 0.000 (-0.002; 0.002) | 0.936 | -0.002 (-0.005; 0.000) | 0.073 |
| C36:3 DAG | -0.003 (-0.006; 0.000) | 0.037 | 0.000 (-0.002; 0.002) | 0.875 | -0.002 (-0.005; 0.000) | 0.050 |
| C36:3 PC | 0.002 (-0.001; 0.005) | 0.142 | 0.001 (-0.001; 0.003) | 0.353 | 0.000 (-0.002; 0.003) | 0.842 |
| C36:3 PC plasmalogen | 0.003 (0.000; 0.006) | 0.028 | 0.001 (-0.001; 0.003) | 0.176 | 0.000 (-0.002; 0.002) | 0.861 |
| C36:3 PE | -0.003 (-0.006; 0.000) | 0.041 | 0.000 (-0.002; 0.002) | 0.970 | -0.002 (-0.004; 0.000) | 0.093 |
| C36:3 PE plasmalogen | 0.000 (-0.002; 0.003) | 0.772 | 0.001 (-0.001; 0.003) | 0.569 | -0.001 (-0.003; 0.002) | 0.609 |
| C36:3 PS plasmalogen | -0.001 (-0.004; 0.002) | 0.345 | -0.001 (-0.003; 0.001) | 0.516 | 0.000 (-0.003; 0.002) | 0.926 |
| C36:4 DAG | -0.008 (-0.011; -0.005) | **<0.001** | -0.002 (-0.004; 0.000) | 0.075 | -0.003 (-0.005; -0.001) | 0.013 |
| C36:4 PC plasmalogen | 0.005 (0.002; 0.008) | **<0.001** | 0.002 (0.000; 0.004) | 0.044 | 0.000 (-0.002; 0.003) | 0.732 |
| C36:4 PCA | -0.003 (-0.006; 0.000) | 0.039 | 0.000 (-0.003; 0.002) | 0.634 | -0.001 (-0.004; 0.001) | 0.259 |
| C36:4 PCB | -0.002 (-0.005; 0.001) | 0.167 | -0.001 (-0.003; 0.001) | 0.537 | -0.001 (-0.003; 0.002) | 0.665 |
| C36:4 PE | -0.003 (-0.006; -0.001) | 0.021 | -0.001 (-0.003; 0.001) | 0.214 | 0.000 (-0.003; 0.002) | 0.726 |
| C36:4 PE plasmalogen | 0.001 (-0.002; 0.004) | 0.504 | 0.001 (-0.001; 0.003) | 0.304 | -0.001 (-0.003; 0.001) | 0.436 |
| C36:5 PC plasmalogenA | 0.006 (0.003; 0.009) | **<0.001** | 0.002 (0.000; 0.004) | 0.016 | 0.000 (-0.002; 0.003) | 0.697 |
| C36:5 PC plasmalogenB | -0.001 (-0.004; 0.002) | 0.397 | 0.000 (-0.002; 0.002) | 0.701 | -0.002 (-0.004; 0.001) | 0.154 |
| C36:5 PE plasmalogen | -0.001 (-0.004; 0.002) | 0.558 | 0.000 (-0.002; 0.002) | 0.779 | -0.001 (-0.004; 0.001) | 0.325 |
| C38:2 PC | 0.003 (0.000; 0.006) | 0.038 | 0.000 (-0.002; 0.002) | 0.840 | 0.002 (0.000; 0.004) | 0.081 |
| C38:2 PE | -0.001 (-0.004; 0.002) | 0.406 | 0.000 (-0.002; 0.002) | 0.737 | -0.002 (-0.004; 0.001) | 0.201 |
| C38:3 PC | 0.001 (-0.002; 0.003) | 0.656 | -0.001 (-0.003; 0.001) | 0.152 | 0.002 (0.000; 0.005) | 0.035 |
| C38:3 PE plasmalogen | 0.000 (-0.003; 0.003) | 0.885 | 0.001 (-0.001; 0.003) | 0.420 | -0.001 (-0.004; 0.001) | 0.263 |
| C38:4 DAG | -0.001 (-0.004; 0.002) | 0.509 | -0.001 (-0.003; 0.002) | 0.621 | 0.000 (-0.002; 0.002) | 0.995 |
| C38:4 PC | -0.001 (-0.004; 0.001) | 0.331 | -0.001 (-0.003; 0.001) | 0.436 | 0.000 (-0.002; 0.002) | 0.946 |
| C38:4 PC plasmalogen | -0.001 (-0.004; 0.001) | 0.316 | -0.002 (-0.004; 0.000) | 0.129 | 0.001 (-0.001; 0.003) | 0.402 |
| C38:4 PE | -0.004 (-0.007; -0.001) | **0.014** | -0.001 (-0.003; 0.001) | 0.470 | -0.001 (-0.004; 0.001) | 0.253 |
| C38:4 PI | 0.000 (-0.003; 0.003) | 0.822 | 0.000 (-0.002; 0.002) | 0.845 | 0.000 (-0.002; 0.003) | 0.704 |
| C38:5 DAG | -0.003 (-0.006; 0.000) | 0.030 | -0.001 (-0.003; 0.001) | 0.322 | -0.001 (-0.003; 0.002) | 0.497 |
| C38:5 PE | -0.001 (-0.004; 0.002) | 0.610 | 0.000 (-0.002; 0.002) | 0.957 | 0.000 (-0.003; 0.002) | 0.733 |
| C38:5 PE plasmalogen | -0.001 (-0.004; 0.001) | 0.346 | 0.000 (-0.002; 0.002) | 0.660 | -0.002 (-0.004; 0.001) | 0.142 |
| C38:6 PC | 0.006 (0.003; 0.008) | **<0.001** | 0.002 (0.000; 0.004) | 0.022 | 0.000 (-0.002; 0.003) | 0.721 |
| C38:6 PC plasmalogen | 0.006 (0.003; 0.008) | **<0.001** | 0.004 (0.002; 0.005) | **<0.001** | -0.001 (-0.003; 0.001) | 0.293 |
| C38:6 PE | 0.000 (-0.003; 0.003) | 0.830 | 0.001 (-0.002; 0.003) | 0.616 | 0.000 (-0.003; 0.002) | 0.687 |
| C38:6 PE plasmalogen | 0.004 (0.001; 0.006) | **0.012** | 0.003 (0.001; 0.005) | 0.010 | -0.001 (-0.004; 0.001) | 0.286 |
| C38:7 PC plasmalogen | 0.005 (0.002; 0.008) | **<0.001** | 0.003 (0.001; 0.005) | **0.005** | -0.001 (-0.003; 0.002) | 0.533 |
| C38:7 PE plasmalogen | 0.004 (0.001; 0.006) | **0.010** | 0.002 (0.000; 0.004) | 0.066 | 0.000 (-0.003; 0.002) | 0.850 |
| C3-DC-CH3 carnitine | -0.002 (-0.005; 0.001) | 0.190 | -0.002 (-0.004; 0.000) | 0.041 | 0.002 (0.000; 0.004) | 0.118 |
| C4 carnitine | -0.001 (-0.003; 0.002) | 0.677 | 0.001 (-0.002; 0.003) | 0.603 | -0.001 (-0.004; 0.001) | 0.301 |
| C40:10 PC | 0.005 (0.002; 0.008) | **<0.001** | 0.002 (0.000; 0.004) | 0.131 | 0.001 (-0.001; 0.004) | 0.279 |
| C40:6 PC | 0.002 (-0.001; 0.005) | 0.125 | 0.000 (-0.002; 0.002) | 0.991 | 0.002 (-0.001; 0.004) | 0.201 |
| C40:6 PE | 0.000 (-0.003; 0.003) | 0.883 | 0.000 (-0.002; 0.002) | 0.867 | 0.000 (-0.002; 0.002) | 0.979 |
| C40:6 PS | 0.003 (0.000; 0.006) | 0.035 | -0.001 (-0.003; 0.001) | 0.553 | 0.003 (0.001; 0.005) | 0.014 |
| C40:7 PC plasmalogen | 0.005 (0.002; 0.007) | **<0.001** | 0.002 (0.000; 0.004) | 0.024 | 0.000 (-0.002; 0.002) | 0.855 |
| C40:7 PE plasmalogen | 0.002 (-0.001; 0.005) | 0.152 | 0.002 (0.000; 0.004) | 0.095 | -0.001 (-0.003; 0.001) | 0.376 |
| C40:9 PC | 0.006 (0.003; 0.009) | **<0.001** | 0.003 (0.001; 0.005) | 0.010 | 0.000 (-0.002; 0.003) | 0.711 |
| C42:0 TG | -0.002 (-0.004; 0.001) | 0.266 | -0.001 (-0.003; 0.001) | 0.225 | 0.001 (-0.001; 0.003) | 0.426 |
| C42:11 PE plasmalogen | 0.007 (0.004; 0.009) | **<0.001** | 0.004 (0.002; 0.006) | **<0.001** | 0.000 (-0.003; 0.002) | 0.723 |
| C44:0 TG | -0.001 (-0.004; 0.002) | 0.432 | -0.001 (-0.003; 0.001) | 0.196 | 0.001 (-0.001; 0.004) | 0.245 |
| C44:1 TG | -0.001 (-0.004; 0.002) | 0.419 | -0.002 (-0.004; 0.000) | 0.108 | 0.002 (-0.001; 0.004) | 0.149 |
| C44:2 TG | -0.003 (-0.006; 0.000) | 0.059 | -0.002 (-0.004; 0.000) | 0.040 | 0.001 (-0.001; 0.004) | 0.312 |
| C45:1 TG | 0.000 (-0.003; 0.003) | 0.868 | 0.000 (-0.002; 0.002) | 0.902 | 0.001 (-0.002; 0.003) | 0.608 |
| C46:0 TG | 0.000 (-0.003; 0.003) | 0.922 | -0.001 (-0.003; 0.001) | 0.380 | 0.001 (-0.001; 0.004) | 0.248 |
| C46:1 TG | -0.001 (-0.004; 0.002) | 0.588 | -0.002 (-0.004; 0.000) | 0.060 | 0.002 (0.000; 0.005) | 0.050 |
| C46:2 TG | -0.003 (-0.006; 0.000) | 0.054 | -0.003 (-0.005; 0.000) | 0.015 | 0.002 (-0.001; 0.004) | 0.142 |
| C46:3 TG | -0.004 (-0.007; -0.001) | **0.008** | -0.003 (-0.005; -0.001) | 0.009 | 0.001 (-0.001; 0.004) | 0.320 |
| C47:1 TG | 0.001 (-0.002; 0.004) | 0.612 | -0.001 (-0.003; 0.002) | 0.612 | 0.001 (-0.001; 0.004) | 0.245 |
| C48:0 TG | -0.002 (-0.005; 0.000) | 0.106 | -0.002 (-0.004; 0.000) | 0.100 | 0.001 (-0.001; 0.003) | 0.464 |
| C48:1 TG | 0.000 (-0.003; 0.003) | 0.858 | -0.001 (-0.003; 0.001) | 0.266 | 0.002 (0.000; 0.004) | 0.113 |
| C48:2 TG | -0.002 (-0.005; 0.001) | 0.195 | -0.002 (-0.004; 0.000) | 0.018 | 0.002 (0.000; 0.005) | 0.066 |
| C48:3 TG | -0.004 (-0.007; -0.002) | **0.002** | -0.003 (-0.005; -0.001) | **0.001** | 0.002 (-0.001; 0.004) | 0.170 |
| C48:4 TG | -0.006 (-0.009; -0.003) | **<0.001** | -0.004 (-0.006; -0.002) | **<0.001** | 0.001 (-0.001; 0.003) | 0.369 |
| C49:1 TG | 0.001 (-0.002; 0.003) | 0.660 | -0.001 (-0.003; 0.001) | 0.403 | 0.002 (-0.001; 0.004) | 0.150 |
| C49:2 TG | 0.000 (-0.003; 0.003) | 0.977 | -0.001 (-0.003; 0.001) | 0.179 | 0.002 (0.000; 0.004) | 0.093 |
| C49:3 TG | -0.003 (-0.006; 0.000) | 0.066 | -0.002 (-0.004; 0.000) | 0.062 | 0.001 (-0.002; 0.003) | 0.504 |
| C4-OH carnitine | 0.001 (-0.002; 0.004) | 0.458 | 0.000 (-0.002; 0.002) | 0.869 | 0.001 (-0.001; 0.003) | 0.448 |
| C5 carnitine | 0.000 (-0.002; 0.003) | 0.772 | 0.000 (-0.002; 0.002) | 0.997 | 0.000 (-0.002; 0.003) | 0.720 |
| C5:1 carnitine | 0.001 (-0.001; 0.004) | 0.331 | 0.001 (-0.001; 0.003) | 0.334 | -0.001 (-0.003; 0.002) | 0.635 |
| C50:0 TG | -0.004 (-0.006; -0.001) | 0.012 | -0.002 (-0.004; 0.000) | 0.087 | 0.000 (-0.002; 0.002) | 0.982 |
| C50:1 TG | 0.000 (-0.003; 0.002) | 0.742 | -0.001 (-0.003; 0.001) | 0.520 | 0.001 (-0.002; 0.003) | 0.558 |
| C50:2 TG | -0.001 (-0.004; 0.002) | 0.593 | -0.001 (-0.003; 0.001) | 0.287 | 0.001 (-0.001; 0.004) | 0.327 |
| C50:3 TG | -0.005 (-0.008; -0.002) | **0.001** | -0.002 (-0.004; 0.000) | 0.032 | 0.000 (-0.002; 0.002) | 0.965 |
| C50:4 TG | -0.007 (-0.010; -0.004) | **<0.001** | -0.003 (-0.005; -0.001) | **0.003** | -0.001 (-0.003; 0.002) | 0.576 |
| C50:5 TG | -0.005 (-0.008; -0.002) | **<0.001** | -0.003 (-0.005; -0.001) | 0.007 | 0.000 (-0.002; 0.003) | 0.837 |
| C51:0 TG | -0.004 (-0.007; -0.001) | **0.003** | -0.001 (-0.003; 0.001) | 0.224 | -0.001 (-0.004; 0.001) | 0.313 |
| C51:1 TG | -0.003 (-0.006; 0.000) | 0.025 | -0.002 (-0.004; 0.000) | 0.095 | 0.000 (-0.002; 0.002) | 0.984 |
| C51:2 TG | 0.002 (0.000; 0.005) | 0.084 | 0.000 (-0.002; 0.002) | 0.929 | 0.002 (-0.001; 0.004) | 0.191 |
| C51:3 TG | -0.002 (-0.005; 0.000) | 0.088 | -0.001 (-0.003; 0.001) | 0.602 | -0.001 (-0.003; 0.001) | 0.371 |
| C52:0 TG | -0.002 (-0.005; 0.000) | 0.089 | -0.001 (-0.003; 0.001) | 0.377 | 0.000 (-0.003; 0.002) | 0.758 |
| C52:1 TG | 0.000 (-0.003; 0.003) | 0.908 | 0.000 (-0.002; 0.002) | 0.814 | 0.000 (-0.002; 0.002) | 0.884 |
| C52:2 TG | 0.004 (0.001; 0.007) | **0.008** | 0.002 (0.000; 0.004) | 0.073 | 0.000 (-0.002; 0.003) | 0.877 |
| C52:3 TG | -0.001 (-0.004; 0.002) | 0.390 | 0.001 (-0.001; 0.003) | 0.485 | -0.002 (-0.004; 0.000) | 0.121 |
| C52:4 TG | -0.007 (-0.010; -0.004) | **<0.001** | -0.001 (-0.003; 0.001) | 0.227 | -0.003 (-0.005; -0.001) | 0.014 |
| C52:5 TG | -0.007 (-0.010; -0.004) | **<0.001** | -0.002 (-0.004; 0.000) | 0.102 | -0.002 (-0.005; 0.000) | 0.043 |
| C52:6 TG | -0.004 (-0.007; -0.001) | **0.004** | -0.002 (-0.004; 0.000) | 0.090 | 0.000 (-0.003; 0.002) | 0.691 |
| C52:7 TG | -0.003 (-0.006; 0.000) | 0.064 | -0.002 (-0.004; 0.000) | 0.080 | 0.001 (-0.002; 0.003) | 0.581 |
| C53:2 TG | 0.005 (0.002; 0.008) | **0.001** | 0.002 (0.000; 0.004) | 0.026 | 0.000 (-0.003; 0.002) | 0.885 |
| C53:3 TG | -0.002 (-0.005; 0.001) | 0.142 | 0.001 (-0.001; 0.003) | 0.398 | -0.003 (-0.005; -0.001) | 0.016 |
| C54:1 TG | 0.001 (-0.002; 0.004) | 0.615 | 0.000 (-0.002; 0.002) | 0.729 | 0.000 (-0.002; 0.002) | 0.951 |
| C54:10 TG | 0.002 (0.000; 0.005) | 0.101 | 0.001 (-0.001; 0.003) | 0.344 | 0.000 (-0.002; 0.002) | 0.918 |
| C54:2 TG | 0.005 (0.003; 0.008) | **<0.001** | 0.003 (0.001; 0.005) | 0.007 | 0.000 (-0.003; 0.002) | 0.910 |
| C54:3 TG | 0.007 (0.004; 0.010) | **<0.001** | 0.004 (0.002; 0.006) | **<0.001** | -0.001 (-0.003; 0.002) | 0.546 |
| C54:4 TG | -0.001 (-0.004; 0.002) | 0.566 | 0.002 (0.000; 0.004) | 0.096 | -0.003 (-0.005; -0.001) | 0.014 |
| C54:5 TG | -0.005 (-0.008; -0.002) | **0.001** | 0.000 (-0.002; 0.002) | 0.838 | -0.004 (-0.006; -0.001) | 0.003 |
| C54:6 TG | -0.004 (-0.007; -0.001) | **0.006** | -0.001 (-0.003; 0.002) | 0.609 | -0.002 (-0.004; 0.000) | 0.090 |
| C54:7 TG | -0.002 (-0.005; 0.001) | 0.160 | 0.000 (-0.002; 0.002) | 0.748 | -0.001 (-0.003; 0.001) | 0.417 |
| C54:8 TG | -0.002 (-0.005; 0.001) | 0.178 | -0.001 (-0.003; 0.001) | 0.581 | -0.001 (-0.003; 0.002) | 0.568 |
| C54:9 TG | -0.002 (-0.005; 0.001) | 0.156 | -0.001 (-0.003; 0.001) | 0.211 | 0.000 (-0.002; 0.003) | 0.810 |
| C55:2 TG | 0.005 (0.002; 0.008) | **<0.001** | 0.003 (0.000; 0.005) | 0.016 | 0.000 (-0.003; 0.002) | 0.824 |
| C55:3 TG | 0.005 (0.002; 0.007) | **0.002** | 0.003 (0.001; 0.005) | **0.003** | -0.002 (-0.004; 0.001) | 0.217 |
| C56:1 TG | 0.001 (-0.002; 0.004) | 0.514 | 0.000 (-0.002; 0.002) | 0.859 | 0.000 (-0.002; 0.003) | 0.727 |
| C56:2 TG | 0.005 (0.002; 0.008) | **0.001** | 0.002 (0.000; 0.004) | 0.018 | 0.000 (-0.002; 0.002) | 0.920 |
| C56:3 TG | 0.006 (0.004; 0.009) | **<0.001** | 0.004 (0.002; 0.006) | **<0.001** | -0.001 (-0.003; 0.001) | 0.418 |
| C56:4 TG | 0.001 (-0.002; 0.004) | 0.539 | 0.002 (0.000; 0.004) | 0.047 | -0.002 (-0.005; 0.000) | 0.044 |
| C56:5 TG | -0.001 (-0.003; 0.002) | 0.697 | 0.001 (-0.001; 0.003) | 0.357 | -0.002 (-0.004; 0.000) | 0.093 |
| C56:6 TG | -0.001 (-0.004; 0.002) | 0.644 | 0.000 (-0.002; 0.002) | 0.921 | -0.001 (-0.003; 0.002) | 0.655 |
| C56:7 TG | 0.003 (0.000; 0.006) | 0.033 | 0.002 (0.000; 0.004) | 0.131 | 0.000 (-0.003; 0.002) | 0.863 |
| C56:8 TG | -0.001 (-0.004; 0.002) | 0.611 | 0.001 (-0.002; 0.003) | 0.598 | -0.001 (-0.004; 0.001) | 0.230 |
| C56:9 TG | -0.001 (-0.004; 0.001) | 0.323 | 0.000 (-0.002; 0.002) | 0.888 | -0.001 (-0.003; 0.001) | 0.435 |
| C58:10 TG | -0.002 (-0.005; 0.001) | 0.183 | -0.001 (-0.003; 0.002) | 0.625 | -0.001 (-0.003; 0.002) | 0.515 |
| C58:11 TG | 0.000 (-0.003; 0.003) | 0.960 | 0.000 (-0.002; 0.002) | 0.878 | 0.000 (-0.002; 0.003) | 0.885 |
| C58:6 TG | -0.003 (-0.006; 0.000) | 0.028 | -0.001 (-0.003; 0.001) | 0.231 | -0.001 (-0.003; 0.002) | 0.492 |
| C58:7 TG | 0.001 (-0.001; 0.004) | 0.323 | 0.001 (-0.001; 0.003) | 0.586 | 0.000 (-0.002; 0.002) | 0.960 |
| C58:8 TG | 0.007 (0.004; 0.010) | **<0.001** | 0.003 (0.001; 0.005) | 0.011 | 0.001 (-0.001; 0.003) | 0.418 |
| C58:9 TG | 0.004 (0.001; 0.006) | **0.015** | 0.002 (0.000; 0.004) | 0.078 | 0.000 (-0.003; 0.002) | 0.838 |
| C5-DC carnitine | 0.000 (-0.003; 0.003) | 0.904 | 0.000 (-0.002; 0.002) | 0.866 | -0.001 (-0.003; 0.002) | 0.633 |
| C6 carnitine | -0.002 (-0.005; 0.001) | 0.114 | -0.001 (-0.003; 0.001) | 0.583 | -0.001 (-0.004; 0.001) | 0.335 |
| C60:12 TG | 0.002 (-0.001; 0.005) | 0.116 | 0.001 (-0.001; 0.003) | 0.374 | 0.000 (-0.002; 0.002) | 0.926 |
| C7 carnitine | -0.001 (-0.004; 0.002) | 0.552 | 0.001 (-0.001; 0.003) | 0.328 | -0.002 (-0.004; 0.000) | 0.108 |
| C8 carnitine | -0.002 (-0.005; 0.001) | 0.165 | 0.000 (-0.002; 0.002) | 0.730 | -0.001 (-0.004; 0.001) | 0.229 |
| C9 carnitine | -0.001 (-0.003; 0.002) | 0.671 | 0.002 (0.000; 0.004) | 0.117 | -0.003 (-0.005; 0.000) | 0.035 |
| Caffeine | -0.001 (-0.004; 0.002) | 0.440 | -0.002 (-0.004; 0.000) | 0.053 | 0.002 (0.000; 0.005) | 0.060 |
| carnitine | -0.001 (-0.004; 0.002) | 0.528 | 0.000 (-0.002; 0.002) | 0.983 | 0.000 (-0.003; 0.002) | 0.710 |
| Cholesterol | 0.002 (0.000; 0.005) | 0.099 | 0.001 (-0.001; 0.003) | 0.540 | 0.001 (-0.002; 0.003) | 0.648 |
| Choline | 0.001 (-0.002; 0.004) | 0.515 | 0.001 (-0.001; 0.003) | 0.310 | -0.001 (-0.003; 0.002) | 0.652 |
| Citrate | -0.001 (-0.004; 0.002) | 0.415 | 0.000 (-0.002; 0.002) | 0.855 | -0.001 (-0.003; 0.001) | 0.373 |
| Citrulline | 0.001 (-0.002; 0.003) | 0.657 | 0.000 (-0.002; 0.002) | 0.669 | 0.001 (-0.001; 0.004) | 0.323 |
| Cortisol | -0.001 (-0.004; 0.002) | 0.393 | 0.000 (-0.003; 0.002) | 0.660 | 0.000 (-0.002; 0.002) | 0.970 |
| Cortisone | 0.002 (-0.001; 0.004) | 0.295 | 0.000 (-0.002; 0.002) | 0.913 | 0.001 (-0.002; 0.003) | 0.570 |
| Cotinine | -0.003 (-0.005; -0.001) | 0.004 | -0.002 (-0.004; -0.001) | 0.004 | 0.001 (-0.001; 0.003) | 0.197 |
| Creatine | 0.001 (-0.001; 0.004) | 0.296 | 0.001 (-0.001; 0.003) | 0.315 | 0.000 (-0.002; 0.002) | 0.894 |
| Creatinine | 0.001 (-0.002; 0.003) | 0.645 | 0.000 (-0.001; 0.002) | 0.659 | 0.000 (-0.002; 0.002) | 0.859 |
| Cytosine | -0.003 (-0.006; 0.000) | 0.047 | 0.000 (-0.002; 0.002) | 0.891 | -0.002 (-0.004; 0.001) | 0.121 |
| Deoxycortisol | -0.001 (-0.004; 0.002) | 0.579 | 0.001 (-0.001; 0.003) | 0.534 | -0.001 (-0.004; 0.001) | 0.244 |
| Dimethylglycine | 0.001 (-0.002; 0.004) | 0.569 | 0.000 (-0.002; 0.002) | 0.961 | 0.001 (-0.002; 0.003) | 0.497 |
| DMGV | 0.000 (-0.003; 0.002) | 0.784 | 0.000 (-0.002; 0.002) | 0.946 | 0.000 (-0.003; 0.002) | 0.762 |
| Ectoine | -0.001 (-0.004; 0.002) | 0.581 | -0.002 (-0.004; 0.000) | 0.041 | 0.003 (0.000; 0.005) | 0.030 |
| Fructose-glucose-galactose | -0.002 (-0.005; 0.000) | 0.063 | -0.001 (-0.002; 0.001) | 0.437 | -0.001 (-0.003; 0.001) | 0.538 |
| Fumarate-maleate | -0.003 (-0.006; 0.000) | 0.035 | -0.003 (-0.005; -0.001) | **<0.001** | 0.002 (0.000; 0.005) | 0.037 |
| GABA | 0.004 (0.001; 0.007) | 0.012 | 0.003 (0.001; 0.005) | 0.009 | -0.001 (-0.004; 0.001) | 0.280 |
| Gamma-butyrobetaine | 0.002 (0.000; 0.005) | 0.103 | -0.001 (-0.002; 0.001) | 0.585 | 0.002 (0.000; 0.004) | 0.097 |
| GDP | -0.004 (-0.007; -0.001) | 0.011 | -0.002 (-0.004; 0.000) | 0.102 | 0.000 (-0.003; 0.002) | 0.700 |
| Gentisate | 0.001 (-0.002; 0.004) | 0.419 | 0.001 (-0.001; 0.003) | 0.543 | 0.000 (-0.002; 0.003) | 0.750 |
| Glucuronate | 0.000 (-0.003; 0.003) | 0.906 | 0.000 (-0.002; 0.002) | 0.928 | 0.000 (-0.002; 0.003) | 0.761 |
| Glutamate | 0.000 (-0.002; 0.003) | 0.817 | 0.002 (0.000; 0.004) | 0.101 | -0.001 (-0.004; 0.001) | 0.208 |
| Glutamine | -0.002 (-0.004; 0.001) | 0.267 | -0.003 (-0.005; -0.001) | 0.012 | 0.002 (0.000; 0.005) | 0.052 |
| Glycine | -0.001 (-0.003; 0.002) | 0.707 | 0.000 (-0.002; 0.002) | 0.823 | 0.000 (-0.002; 0.002) | 0.918 |
| Glycocholate | 0.000 (-0.003; 0.003) | 0.807 | 0.001 (-0.001; 0.003) | 0.287 | -0.001 (-0.003; 0.001) | 0.406 |
| Glycodeoxycholic acid | 0.002 (-0.001; 0.005) | 0.154 | 0.001 (-0.001; 0.003) | 0.213 | 0.000 (-0.002; 0.002) | 0.922 |
| GMP | -0.004 (-0.007; -0.001) | 0.013 | -0.002 (-0.005; 0.000) | 0.019 | 0.000 (-0.002; 0.003) | 0.699 |
| Guanidoacetic acid | 0.000 (-0.003; 0.002) | 0.971 | -0.002 (-0.003; 0.000) | 0.076 | 0.002 (0.000; 0.004) | 0.039 |
| Guanine | 0.001 (-0.002; 0.004) | 0.581 | 0.000 (-0.002; 0.003) | 0.640 | 0.000 (-0.002; 0.002) | 1.000 |
| Hexose monophosphate | -0.004 (-0.007; -0.001) | **0.007** | -0.003 (-0.005; -0.001) | 0.006 | 0.001 (-0.001; 0.004) | 0.284 |
| Hippurate | 0.002 (-0.001; 0.005) | 0.247 | 0.001 (-0.001; 0.003) | 0.326 | 0.000 (-0.003; 0.002) | 0.807 |
| Histidine | 0.000 (-0.003; 0.003) | 0.870 | -0.001 (-0.003; 0.001) | 0.204 | 0.002 (0.000; 0.004) | 0.119 |
| Homoarginine | 0.005 (0.002; 0.007) | **0.001** | 0.001 (-0.001; 0.003) | 0.527 | 0.002 (0.000; 0.005) | 0.036 |
| Hydroxycotinine | -0.003 (-0.005; 0.000) | 0.029 | -0.002 (-0.003; 0.000) | 0.039 | 0.000 (-0.002; 0.002) | 0.762 |
| hydroxyectoine | 0.001 (-0.002; 0.003) | 0.672 | 0.000 (-0.002; 0.002) | 0.672 | 0.001 (-0.001; 0.003) | 0.336 |
| Hydroxyproline | 0.001 (-0.002; 0.004) | 0.536 | 0.001 (-0.001; 0.003) | 0.207 | -0.002 (-0.004; 0.001) | 0.204 |
| Hypoxanthine | 0.004 (0.001; 0.007) | **0.006** | 0.000 (-0.002; 0.002) | 0.826 | 0.003 (0.000; 0.005) | 0.019 |
| Indole-3-propionate | 0.000 (-0.003; 0.003) | 0.788 | -0.001 (-0.003; 0.001) | 0.190 | 0.002 (0.000; 0.005) | 0.082 |
| Indoxylsulfate | 0.002 (-0.001; 0.005) | 0.277 | 0.000 (-0.002; 0.002) | 0.739 | 0.001 (-0.002; 0.003) | 0.581 |
| Inosine | 0.001 (-0.002; 0.004) | 0.474 | 0.000 (-0.002; 0.002) | 0.847 | 0.001 (-0.002; 0.003) | 0.681 |
| Inosine | 0.001 (-0.002; 0.004) | 0.474 | 0.000 (-0.002; 0.002) | 0.847 | 0.001 (-0.002; 0.003) | 0.681 |
| Inositol | 0.001 (-0.002; 0.004) | 0.544 | 0.002 (0.000; 0.004) | 0.124 | -0.001 (-0.004; 0.001) | 0.281 |
| Isocitrate | 0.000 (-0.002; 0.003) | 0.754 | 0.000 (-0.002; 0.002) | 0.885 | 0.000 (-0.002; 0.003) | 0.709 |
| Isoleucine | 0.001 (-0.001; 0.004) | 0.369 | 0.000 (-0.002; 0.002) | 0.785 | 0.001 (-0.001; 0.003) | 0.499 |
| Kynurenic acid | 0.000 (-0.003; 0.003) | 0.837 | 0.000 (-0.002; 0.002) | 0.902 | -0.001 (-0.003; 0.002) | 0.647 |
| Kynurenine | 0.001 (-0.002; 0.004) | 0.482 | -0.001 (-0.003; 0.001) | 0.467 | 0.002 (0.000; 0.004) | 0.081 |
| Lactate | 0.002 (-0.001; 0.005) | 0.221 | -0.001 (-0.003; 0.001) | 0.177 | 0.004 (0.001; 0.006) | **0.002** |
| Lactose | -0.005 (-0.007; -0.002) | **0.001** | -0.003 (-0.005; -0.001) | 0.010 | 0.000 (-0.002; 0.003) | 0.725 |
| Leucine | 0.002 (-0.001; 0.005) | 0.191 | 0.000 (-0.002; 0.002) | 0.810 | 0.001 (-0.001; 0.003) | 0.317 |
| Linoleoylethanolamide | 0.001 (-0.002; 0.004) | 0.568 | 0.000 (-0.003; 0.002) | 0.655 | 0.001 (-0.002; 0.003) | 0.654 |
| Lysine | 0.003 (0.000; 0.006) | 0.064 | 0.001 (-0.001; 0.003) | 0.405 | 0.001 (-0.002; 0.003) | 0.516 |
| Malate | -0.002 (-0.005; 0.001) | 0.145 | -0.004 (-0.006; -0.002) | **<0.001** | 0.004 (0.002; 0.006) | **<0.001** |
| Methionine | 0.000 (-0.003; 0.003) | 0.913 | 0.000 (-0.002; 0.002) | 0.685 | 0.000 (-0.002; 0.003) | 0.690 |
| Methionine sulfoxide | 0.000 (-0.003; 0.002) | 0.762 | 0.000 (-0.002; 0.002) | 0.940 | 0.000 (-0.002; 0.003) | 0.880 |
| Myristoleic acid | 0.001 (-0.002; 0.004) | 0.373 | 0.000 (-0.002; 0.002) | 0.909 | 0.001 (-0.001; 0.003) | 0.377 |
| N1-Acetylspermidine | 0.000 (-0.003; 0.003) | 0.916 | 0.001 (-0.001; 0.003) | 0.276 | -0.001 (-0.004; 0.001) | 0.329 |
| N1-methyl-2-pyridone-5-carboxamide | 0.000 (-0.003; 0.003) | 0.817 | 0.001 (-0.001; 0.003) | 0.282 | -0.001 (-0.004; 0.001) | 0.281 |
| N4-Acetylcytidine | -0.002 (-0.005; 0.001) | 0.150 | -0.001 (-0.003; 0.001) | 0.327 | 0.000 (-0.002; 0.002) | 0.913 |
| N6-Acetyllysine | 0.000 (-0.003; 0.003) | 0.821 | 0.001 (-0.001; 0.003) | 0.500 | 0.000 (-0.003; 0.002) | 0.732 |
| N-Acetylaspartic acid | 0.001 (-0.002; 0.003) | 0.714 | 0.001 (-0.001; 0.003) | 0.566 | 0.000 (-0.003; 0.002) | 0.778 |
| N-Acetylornithine | 0.000 (-0.003; 0.003) | 0.855 | 0.001 (-0.001; 0.003) | 0.495 | 0.000 (-0.003; 0.002) | 0.686 |
| N-Acetylputrescine | -0.003 (-0.005; 0.000) | 0.075 | -0.001 (-0.003; 0.001) | 0.424 | 0.000 (-0.003; 0.002) | 0.751 |
| N-alpha-Acetyl-L-arginine | 0.001 (-0.002; 0.003) | 0.721 | -0.001 (-0.003; 0.002) | 0.634 | 0.001 (-0.001; 0.003) | 0.405 |
| N-carbamoyl-beta-alanine | 0.000 (-0.003; 0.003) | 0.871 | 0.000 (-0.002; 0.002) | 0.839 | 0.001 (-0.002; 0.003) | 0.573 |
| Niacinamide | -0.001 (-0.004; 0.002) | 0.578 | -0.001 (-0.003; 0.001) | 0.498 | 0.000 (-0.002; 0.003) | 0.681 |
| N-Methyl-L-proline | 0.000 (-0.003; 0.003) | 0.907 | 0.000 (-0.002; 0.002) | 0.815 | -0.001 (-0.003; 0.002) | 0.605 |
| N-methylmalonic acid | 0.003 (0.000; 0.006) | 0.044 | 0.001 (-0.001; 0.003) | 0.281 | 0.001 (-0.001; 0.003) | 0.396 |
| N-oleoyl glycine | 0.004 (0.001; 0.007) | 0.005 | 0.001 (-0.001; 0.003) | 0.455 | 0.002 (-0.001; 0.004) | 0.131 |
| Ornithine | 0.006 (0.003; 0.009) | **<0.001** | 0.000 (-0.002; 0.002) | 0.712 | 0.005 (0.002; 0.007) | **<0.001** |
| Oxalate | -0.001 (-0.004; 0.001) | 0.341 | -0.001 (-0.003; 0.001) | 0.374 | 0.000 (-0.003; 0.002) | 0.913 |
| Pantothenate | 0.002 (-0.001; 0.005) | 0.270 | 0.003 (0.001; 0.005) | **0.003** | -0.003 (-0.005; -0.001) | 0.011 |
| Pantothenol | 0.000 (-0.003; 0.003) | 0.963 | 0.001 (-0.001; 0.003) | 0.512 | 0.000 (-0.003; 0.002) | 0.705 |
| Phenylacetylglutamine | 0.000 (-0.003; 0.003) | 0.967 | 0.000 (-0.002; 0.002) | 0.976 | 0.000 (-0.002; 0.002) | 0.972 |
| Phenylalanine | 0.000 (-0.003; 0.003) | 0.904 | 0.000 (-0.002; 0.002) | 0.831 | 0.001 (-0.002; 0.003) | 0.651 |
| Phosphocholine | 0.000 (-0.003; 0.002) | 0.759 | 0.001 (-0.001; 0.003) | 0.593 | -0.001 (-0.003; 0.002) | 0.519 |
| Phosphocreatine | 0.001 (-0.002; 0.004) | 0.551 | 0.000 (-0.002; 0.002) | 0.691 | 0.001 (-0.002; 0.003) | 0.579 |
| Phosphoglycerate | -0.004 (-0.007; -0.001) | **0.003** | 0.000 (-0.003; 0.002) | 0.640 | -0.002 (-0.005; 0.000) | 0.046 |
| Pipecolic acid | 0.000 (-0.003; 0.003) | 0.957 | 0.001 (-0.001; 0.003) | 0.259 | -0.001 (-0.004; 0.001) | 0.214 |
| Piperine | -0.003 (-0.006; 0.000) | 0.053 | 0.002 (0.000; 0.004) | 0.025 | -0.005 (-0.007; -0.003) | **<0.001** |
| Proline | -0.003 (-0.006; 0.000) | 0.072 | -0.003 (-0.005; -0.001) | 0.010 | 0.002 (0.000; 0.004) | 0.121 |
| Proline betaine | 0.000 (-0.003; 0.003) | 0.909 | 0.001 (-0.001; 0.003) | 0.471 | -0.001 (-0.004; 0.001) | 0.346 |
| Pseudouridine | 0.000 (-0.003; 0.002) | 0.728 | 0.000 (-0.002; 0.002) | 0.913 | 0.000 (-0.003; 0.002) | 0.751 |
| Pyroglutamic acid | -0.002 (-0.005; 0.001) | 0.252 | -0.003 (-0.005; -0.001) | 0.012 | 0.002 (0.000; 0.005) | 0.050 |
| Quinolinate | -0.001 (-0.004; 0.002) | 0.524 | 0.000 (-0.002; 0.002) | 0.831 | -0.001 (-0.003; 0.002) | 0.532 |
| Ribothymidine | 0.001 (-0.002; 0.004) | 0.382 | 0.000 (-0.002; 0.002) | 0.939 | 0.001 (-0.001; 0.003) | 0.414 |
| Salicylurate | 0.000 (-0.003; 0.003) | 0.911 | 0.000 (-0.002; 0.002) | 0.855 | 0.000 (-0.002; 0.003) | 0.880 |
| Sarcosine | -0.002 (-0.004; 0.001) | 0.257 | 0.001 (-0.001; 0.003) | 0.481 | -0.002 (-0.004; 0.000) | 0.098 |
| SDMA | 0.002 (-0.001; 0.004) | 0.231 | 0.000 (-0.002; 0.002) | 0.692 | 0.001 (-0.001; 0.003) | 0.433 |
| Serine | 0.000 (-0.003; 0.003) | 0.886 | -0.001 (-0.003; 0.001) | 0.570 | 0.001 (-0.001; 0.003) | 0.417 |
| Sorbitol | -0.002 (-0.005; 0.001) | 0.194 | -0.001 (-0.003; 0.001) | 0.297 | 0.000 (-0.002; 0.003) | 0.795 |
| Sphinganine | -0.001 (-0.004; 0.002) | 0.589 | -0.004 (-0.006; -0.001) | <0.001 | 0.004 (0.001; 0.006) | 0.003 |
| Sphingosine | 0.005 (0.002; 0.007) | **0.002** | -0.001 (-0.003; 0.001) | 0.285 | 0.005 (0.002; 0.007) | **<0.001** |
| Suberate | 0.000 (-0.003; 0.003) | 0.791 | -0.001 (-0.003; 0.001) | 0.223 | 0.002 (0.000; 0.005) | 0.044 |
| Succinate | 0.000 (-0.003; 0.003) | 0.926 | 0.000 (-0.002; 0.002) | 0.856 | 0.000 (-0.002; 0.002) | 0.995 |
| Sucrose | -0.003 (-0.006; 0.000) | 0.053 | -0.002 (-0.004; 0.000) | 0.020 | 0.001 (-0.001; 0.004) | 0.229 |
| Taurine | -0.002 (-0.005; 0.001) | 0.184 | -0.001 (-0.003; 0.001) | 0.206 | 0.000 (-0.002; 0.003) | 0.681 |
| Taurodeoxycholate-taurochenodeoxycholate | 0.001 (-0.002; 0.004) | 0.610 | 0.000 (-0.002; 0.002) | 0.917 | 0.001 (-0.002; 0.003) | 0.596 |
| Thiamine | 0.000 (-0.003; 0.003) | 0.878 | 0.001 (-0.001; 0.003) | 0.572 | 0.000 (-0.003; 0.002) | 0.763 |
| Threonine | -0.001 (-0.004; 0.001) | 0.324 | -0.001 (-0.003; 0.001) | 0.248 | 0.000 (-0.002; 0.003) | 0.820 |
| Thyroxine | -0.002 (-0.004; 0.001) | 0.295 | 0.000 (-0.002; 0.002) | 0.776 | -0.001 (-0.003; 0.001) | 0.360 |
| TMAO | 0.002 (-0.001; 0.004) | 0.310 | 0.002 (0.000; 0.004) | 0.035 | -0.002 (-0.005; 0.000) | 0.056 |
| Trigonelline (N-methylnicotinate) | 0.000 (-0.003; 0.003) | 0.866 | -0.001 (-0.003; 0.001) | 0.343 | 0.002 (-0.001; 0.004) | 0.169 |
| Trimethylbenzene | 0.002 (-0.001; 0.005) | 0.240 | 0.001 (-0.001; 0.003) | 0.227 | 0.000 (-0.003; 0.002) | 0.751 |
| Trimethyllysine | 0.003 (0.001; 0.006) | 0.020 | 0.002 (0.000; 0.004) | 0.024 | -0.001 (-0.003; 0.001) | 0.440 |
| Tryptophan | 0.004 (0.001; 0.007) | 0.003 | 0.002 (0.000; 0.004) | 0.029 | 0.000 (-0.002; 0.002) | 0.894 |
| Tyrosine | 0.000 (-0.003; 0.003) | 0.911 | -0.001 (-0.003; 0.001) | 0.598 | 0.001 (-0.001; 0.003) | 0.363 |
| UDP | -0.002 (-0.005; 0.001) | 0.127 | 0.000 (-0.002; 0.002) | 0.827 | -0.001 (-0.004; 0.001) | 0.256 |
| Uracil | 0.002 (-0.001; 0.005) | 0.268 | 0.001 (-0.001; 0.003) | 0.449 | 0.000 (-0.002; 0.003) | 0.904 |
| Urate | 0.002 (-0.001; 0.005) | 0.147 | 0.002 (0.000; 0.004) | 0.058 | -0.001 (-0.003; 0.001) | 0.385 |
| Uric acid | 0.002 (-0.001; 0.005) | 0.151 | 0.001 (-0.001; 0.003) | 0.250 | 0.000 (-0.002; 0.002) | 0.991 |
| Uridine | 0.001 (-0.002; 0.004) | 0.372 | 0.001 (-0.001; 0.003) | 0.388 | 0.000 (-0.003; 0.002) | 0.844 |
| Urocanic acid | 0.000 (-0.003; 0.003) | 0.985 | 0.000 (-0.002; 0.002) | 0.831 | 0.000 (-0.002; 0.003) | 0.900 |
| Valine | 0.004 (0.001; 0.007) | 0.011 | 0.002 (0.000; 0.004) | 0.084 | 0.000 (-0.002; 0.003) | 0.813 |
| Xanthine | 0.001 (-0.002; 0.004) | 0.448 | 0.000 (-0.002; 0.002) | 0.734 | 0.001 (-0.001; 0.004) | 0.289 |
| Xanthine | 0.001 (-0.002; 0.004) | 0.448 | 0.000 (-0.002; 0.002) | 0.734 | 0.001 (-0.001; 0.004) | 0.289 |
| Xanthosine | 0.001 (-0.001; 0.004) | 0.344 | 0.001 (0.000; 0.003) | 0.137 | -0.001 (-0.003; 0.001) | 0.353 |
| Multivariable linear regression models adjusted for recruiting center, smoking status, sex, BMI, age, education, and physical activity (METs/day).  Abbreviations: 95% CI, 95% confident interval; AAMU, 5-acetamido-6-amino-3-methyl uracil; ADP, Adenosine diphosphate; BMI, body mass index; CE, cholesterol ester; DAG, diacylglycerol; DMGV, dimethylguanidino valeric acid; LPC, Lysophosphatidylcholine; LPE, lysophosphatidylethanolamine; MAG, monoacylglycerol; PC, phosphatidylcholine; PS, phosphatidylserine; SM, Sphingomyelin; TAG, triacylglycerol; TMAO, Trimethylamine N-oxide.  Significant metabolites associated with olive oil consumption after FDR (< 0.05) are indicated in bold. | | | | | | |

**Additional Table 2**. Hazard ratios (95% CIs) of type 2 diabetes incidence according to metabolomics profiles of energy-adjusted olive oil and its subtypes in the PREDIMED study groups stratified by intervention group.

|  |  | Type 2 diabetes | | | |
| --- | --- | --- | --- | --- | --- |
|  |  | Baseline visit | | 1-year visit | |
| MedDiet+EVOO group | Baseline Consumption (g/day) | HR (95% CI) | *P* | HR (95% CI) | *P* |
| Cases/total participants |  | 75/282 |  | 50/229 |  |
|  |  |  |  |  |  |
| Total olive oil | 40 ± 18 | 1.41 (0.86, 2.33) | 0.173 | 0.83 (0.48, 1.42) | 0.497 |
| Extra virgin olive oil | 22 ± 24 | 1.48 (0.90, 2.41) | 0.122 | 0.85 (0.48, 1.52) | 0.594 |
| Common olive oil | 19 ± 21 | 0.70 (0.45, 1.11) | 0.129 | 0.99 (0.64, 1.53) | 0.954 |
|  |  |  |  |  |  |
| MedDiet+Nuts group |  | HR (95% CI) | *P* | HR (95% CI) | *P* |
| Cases/total participants |  | 82/335 |  | 51/249 |  |
|  |  |  |  |  |  |
| Total olive oil | 39 ± 18 | 0.92 (0.66, 1.28) | 0.620 | 1.25 (0.69, 2.24) | 0.461 |
| Extra virgin olive oil | 24 ± 24 | 0.98 (0.64, 1.48) | 0.914 | 1.15 (0.73, 1.83) | 0.550 |
| Common olive oil | 15 ± 18 | 0.80 (0.53, 1.20) | 0.282 | 0.96 (0.59, 1.58) | 0.882 |
|  |  |  |  |  |  |
| Control group |  | HR (95% CI) | P | HR (95% CI) | P |
| Cases/total participants |  | 88/306 |  | 60/226 |  |
|  |  |  |  |  |  |
| Total olive oil | 39 ± 18 | 0.99 (0.71, 1.38) | 0.935 | 0.90 (0.53, 1.54) | 0.707 |
| Extra virgin olive oil | 20 ± 23 | 0.83 (0.55, 1.25) | 0.382 | 0.87 (0.50, 1.50) | 0.605 |
| Common olive oil | 19 ± 21 | 1.25 (0.88, 1.78) | 0.209 | 0.85 (0.54, 1.33) | 0.479 |

Models adjusted for age (years), sex, propensity scores, BMI, smoking status (never, former, or current smoker), alcohol intake and squared alcohol intake (g/day), education level (primary, secondary, academic), physical activity (metabolic-equivalent minutes per day), family history of CHD (yes/no), dyslipidemia, hypertension, dyslipidemia, and hypertension treatment, intakes of vegetables, fruits, cereals, nuts, eggs, legumes, meat, fish, and dairy(g/day); and stratified by recruitment center. Abbreviations: HR, hazard ratio; CI, confidence interval; CHD, coronary heart disease; BMI, body mass index.

**Additional Table 3**. Hazard ratios (95% CIs) of cardiovascular disease incidence according to metabolomics profiles of energy-adjusted olive oil and its subtypes in the PREDIMED study groups stratified by intervention group.

|  |  | Cardiovascular diseases | | | |
| --- | --- | --- | --- | --- | --- |
|  |  | Baseline visit | | 1-year visit | |
| MedDiet+EVOO group | Baseline Consumption (g/day) | HR (95% CI) | *P* | HR (95% CI) | *P* |
| Cases/total participants |  | 81/368 |  | 65/349 |  |
|  |  |  |  |  |  |
| Total olive oil | 41 ± 18 | 0.62 (0.47, 0.82) | <0.001 | 0.71 (0.53, 0.97) | 0.030 |
| Extra virgin olive oil | 22 ± 24 | 0.50 (0.37, 0.69) | <0.001 | 0.51 (0.36, 0.75) | <0.001 |
| Common olive oil | 19 ± 21 | 1.78 (1.31, 2.41) | <0.001 | 1.65 (1.09, 2.51) | 0.018 |
|  |  |  |  |  |  |
| MedDiet+Nuts group |  | HR (95% CI) | *P* | HR (95% CI) | *P* |
| Cases/total participants |  | 64/318 |  | 43/293 |  |
|  |  |  |  |  |  |
| Total olive oil | 39 ± 18 | 0.77 (0.55, 1.08) | 0.125 | 0.97 (0.63, 1.52) | 0.905 |
| Extra virgin olive oil | 24 ± 24 | 0.75 (0.54, 1.05) | 0.095 | 0.90 (0.59, 1.41) | 0.671 |
| Common olive oil | 15 ± 18 | 1.59 (1.10, 2.31) | 0.015 | 1.42 (0.81, 2.49) | 0.223 |
|  |  |  |  |  |  |
| Control group |  | HR (95% CI) | *P* | HR (95% CI) | *P* |
| Cases/total participants |  | 77/307 |  | 51/274 |  |
|  |  |  |  |  |  |
| Total olive oil | 39 ± 18 | 0.79 (0.56, 1.11) | 0.175 | 1.26 (0.86, 1.86) | 0.242 |
| Extra virgin olive oil | 20 ± 23 | 0.70 (0.50, 0.98) | 0.040 | 1.19 (0.80, 1.78) | 0.387 |
| Common olive oil | 19 ± 21 | 0.96 (0.68, 1.37) | 0.828 | 0.70 (0.45, 1.08) | 0.107 |
| Models adjusted for age (years), sex, propensity scores, BMI, smoking status (never, former, or current smoker), alcohol intake and squared alcohol intake (g/day), education level (primary, secondary, academic), physical activity (metabolic-equivalent minutes per day), family history of CHD (yes/no), dyslipidemia, hypertension, dyslipidemia, and hypertension treatment, intakes of vegetables, fruits, cereals, nuts, eggs, legumes, meat, fish, and dairy (g/day); and stratified by recruitment center. (g/day). Abbreviations: HR, hazard ratio; CI, confidence interval; CHD, coronary heart disease; BMI, body mass index. | | | | | |

**Additional Figure 1**. Flowchart of participants and analysis.

Abbreviations: PREDIMED, PREvención con DIeta MEDiterranea study; CVD, cardiovascular disease; T2D, type 2 diabetes; OGTT, oral glucose tolerance test; FFQ, food frequency questionnaire; CV, cross-validation; ENR, elastic net regression.


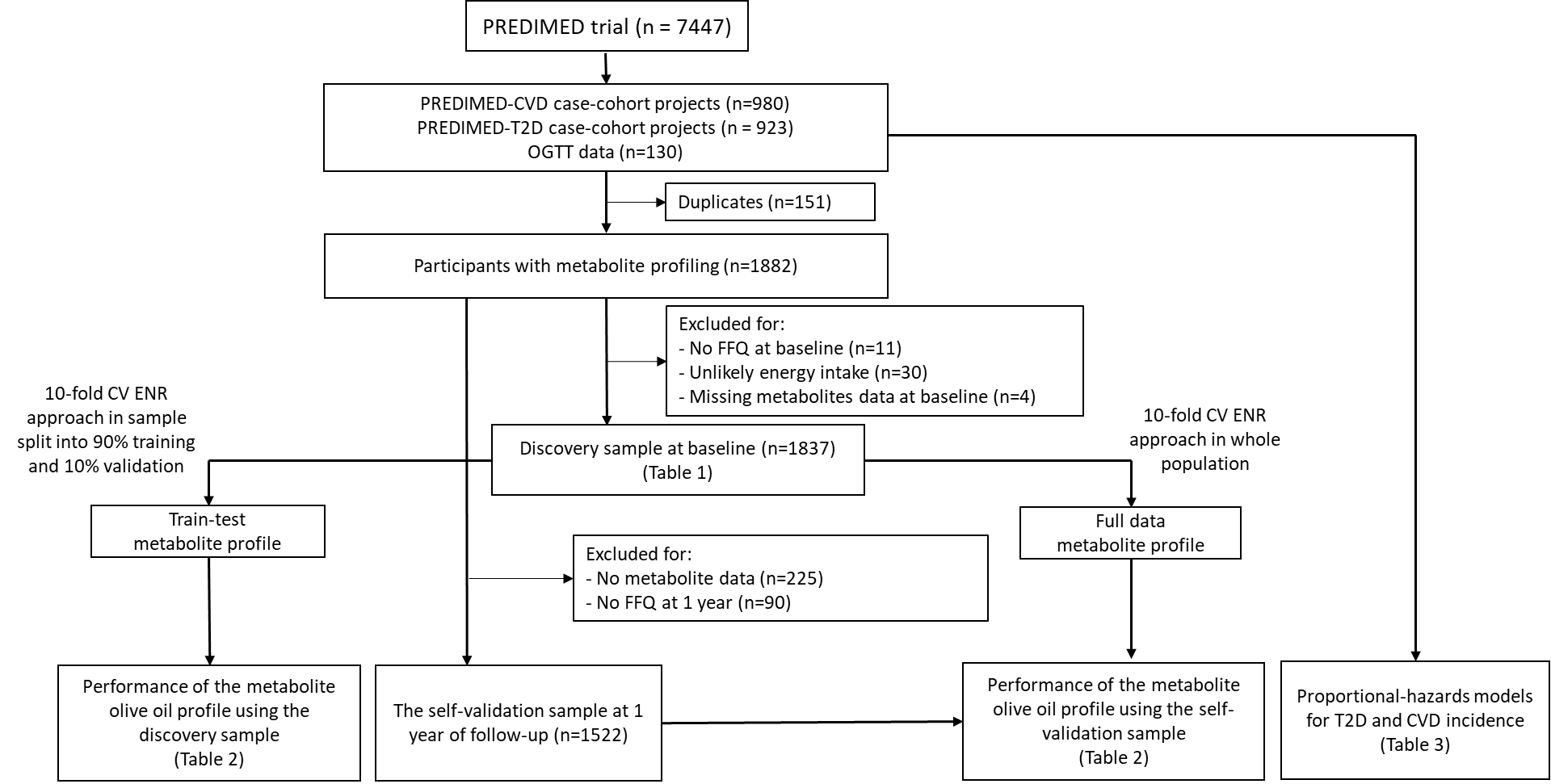


**Supplementary Figure 2**. Volcano plot showing the associations between plasma metabolites and common olive oil consumption at baseline.

The models were adjusted by recruiting center, smoking status (former smoker, never smoker, smoker), sex, BMI, age, education, and physical activity (METs/day). An FDR <0.05 was considered statistically significant (up dotted line).


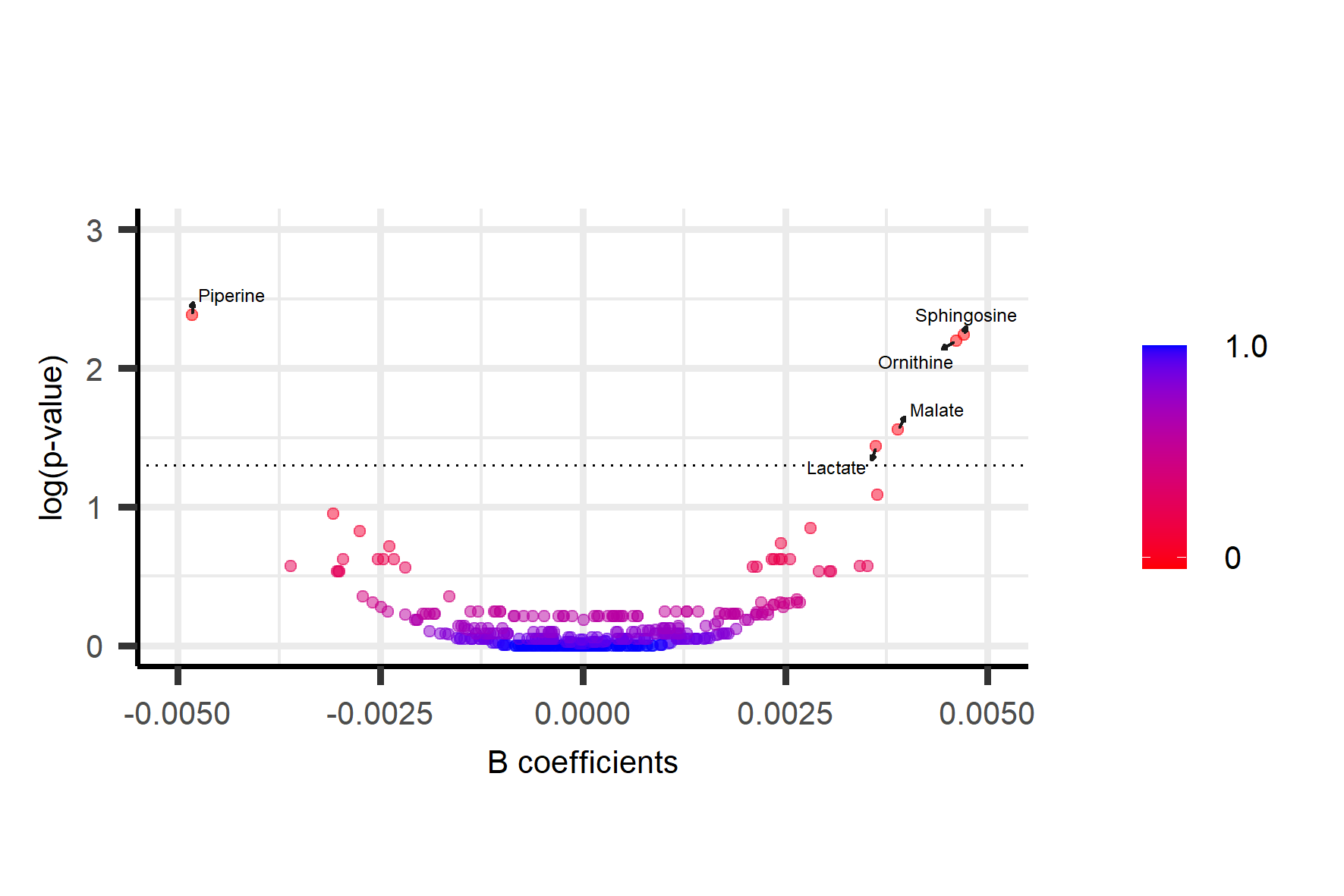


**Additional Figure 3**. Metabolite coefficients (mean and SD) selected ten times in the 10-cross validation of the continuous elastic regression for energy-adjusted common olive oil.

The sets of metabolites were selected using elastic continuous regression models (with lambda.min) employing the whole dataset of subjects (n=1833). Negative coefficients are plotted on the left, whereas positive coefficients are shown on the right.


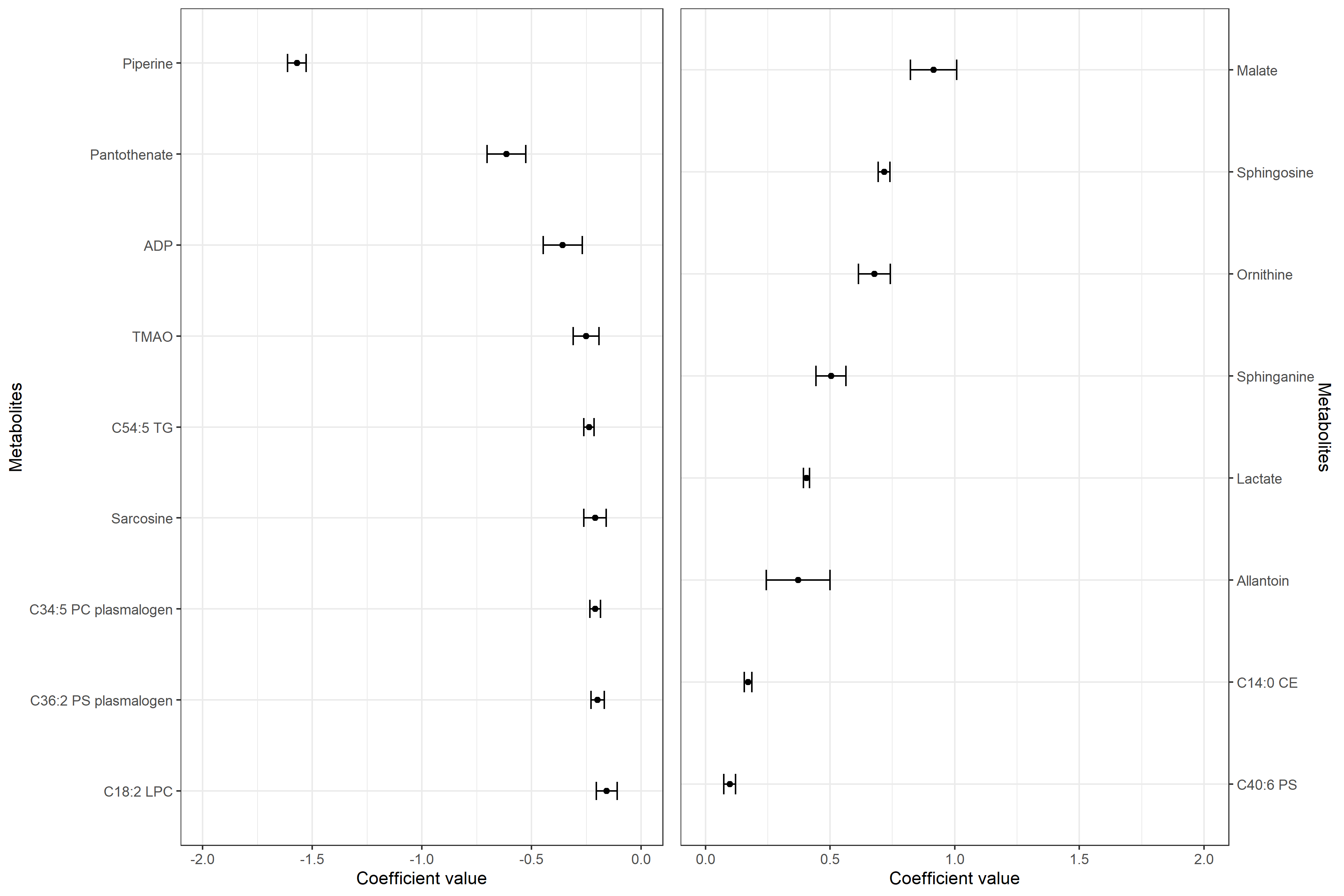


**Additional Figure 4**. Biplot of the principal component analysis using metabolites’ coefficients derived by the elastic net continuous regression of each olive oil consumption approach.

This biplot includes the two first principal components using the three metabolomic profiles (total olive oil, EVOO, and COO) as the “variables” and the different metabolites as the “individuals”. The intensity of the metabolite arrow reflects its overall contribution to principal components 1 and 2 (from gray (low contribution) to black (high contribution)). Metabolites’ names are colored according to their quality in the factor map (from blue (low quality) to red (high quality)). The top 30 metabolites that make the most significant contribution are presented to facilitate understanding of the graph. Abbreviations: ADP, Adenosine diphosphate; CAR, Carnitine; CE, Cholesterol ester; Contrib, Contribution; COO, Common Olive Oil; Dim, Dimension; EVOO, Extra-Virgin Olive Oil; LPE, Lyso phosphatidyl ethanolamine; PC, Phosphatidylcholine; plas, Plasminogen; TAG, Triacylglycerol; Vit, Vitamin.


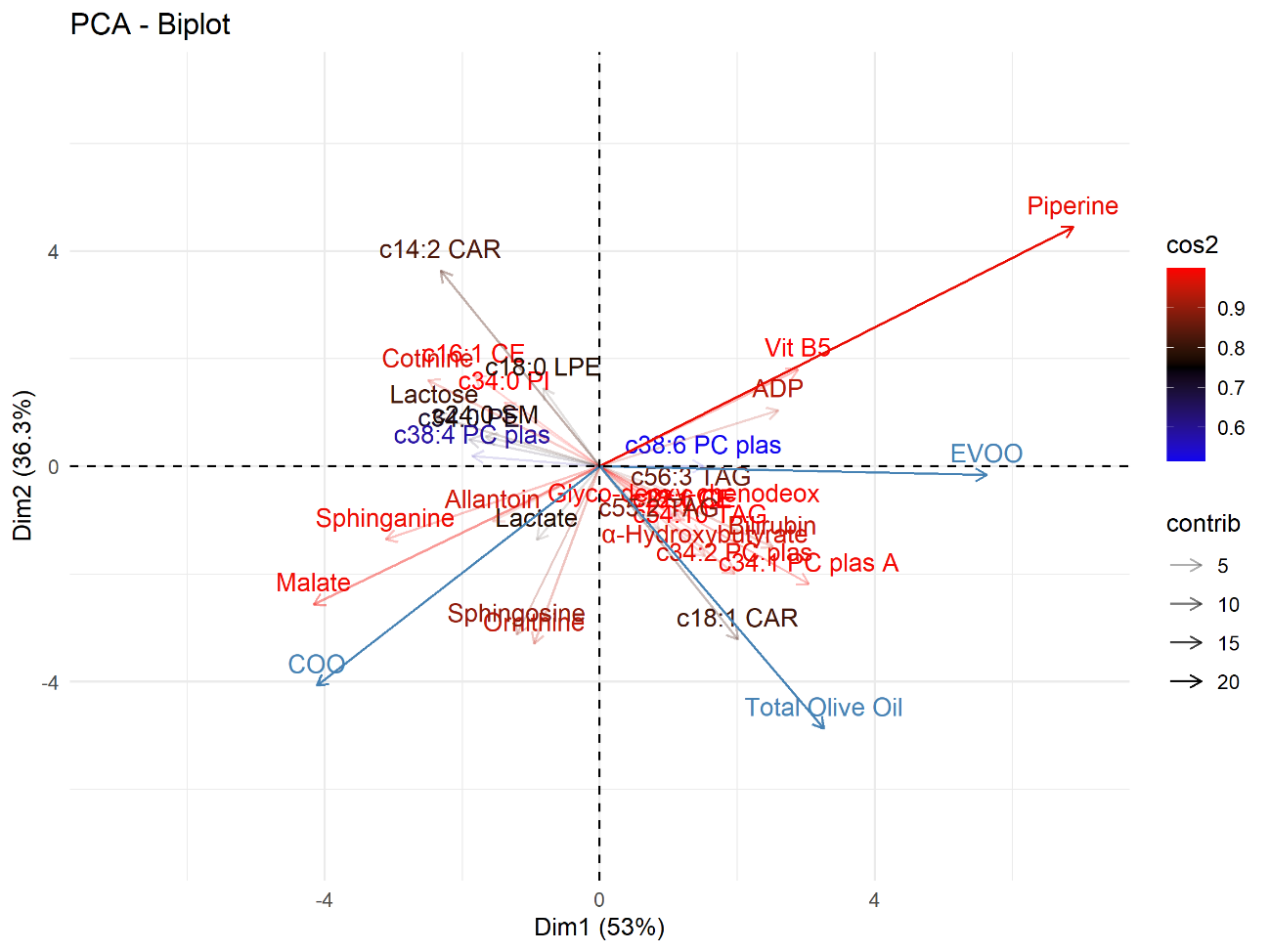


**Additional Figure 5**. Correlation plot showing the correlation between each self-reported olive oil intake and olive oil metabolomics profiles and each food group consumption.

Abbreviations: TOO, total olive oil; EVOO, extra virgin olive oil; COO, common olive oil; mp, metabolomics profile.


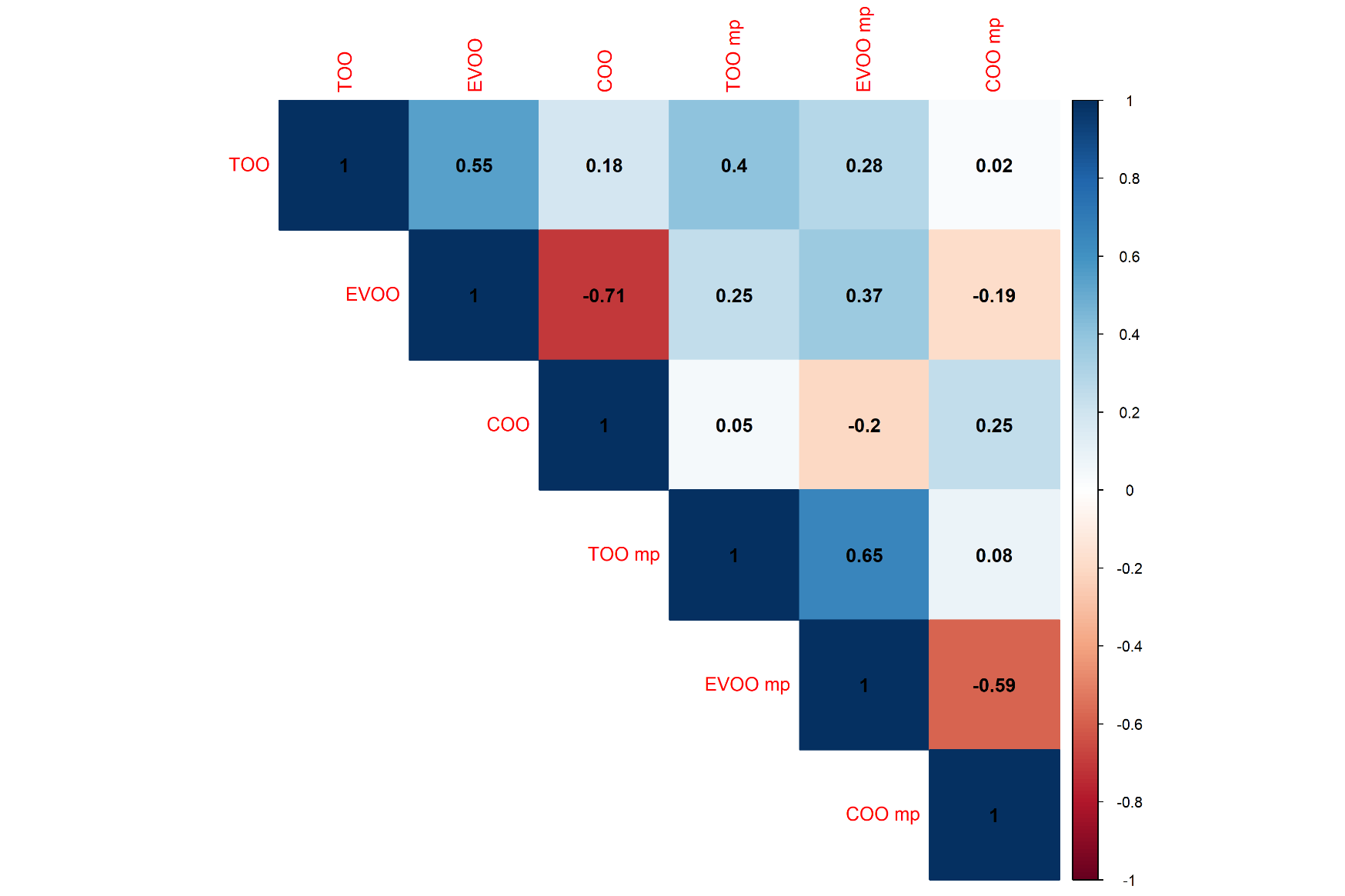

Supplement: Supplementary file 1 — Additional file1: Table S1. Associations between plasma metabolites and total olive oil, extra virgin olive oil, and common olive oil consumption at baseline. Table S2. Hazard ratios (95% CIs) of type 2 diabetes incidence according to metabolomics profiles of energy-adjusted olive oil and its subtypes in the PREDIMED study groups stratified by intervention group. Table S3. Hazard ratios (95% CIs) of cardiovascular disease incidence according to metabolomics profiles of energy-adjusted olive oil and its subtypes in the PREDIMED study groups stratified by intervention group. Figure S1. Flowchart of participants and analysis. Figure S2. Volcano plot showing the associations between plasma metabolites and common olive oil consumption at baseline. Figure S3. Metabolite coefficients (mean and SD) selected ten times in the 10-cross validation of the continuous elastic regression for energy-adjusted common olive oil. Figure S4. Biplot of the principal component analysis using metabolites’ coefficients derived by the elastic net continuous regression of each olive oil consumption approach. Figure S5. Correlation plot showing the correlation between each self-reported olive oil intake and olive oil metabolomics profiles and each food group consumption. [file 12933_2023_2066_MOESM1_ESM.docx]
